# Faecal microbiota transplantation halts progression of human new-onset type 1 diabetes in a randomised controlled trial

**DOI:** 10.1136/gutjnl-2020-322630

**Published:** 2020-10-26

**Authors:** Pieter de Groot, Tanja Nikolic, Silvia Pellegrini, Valeria Sordi, Sultan Imangaliyev, Elena Rampanelli, Nordin Hanssen, Ilias Attaye, Guido Bakker, Gaby Duinkerken, Antoinette Joosten, Andrei Prodan, Evgeni Levin, Han Levels, Bartjan Potter van Loon, Arianne van Bon, Catherina Brouwer, Sytze van Dam, Suat Simsek, Daniel van Raalte, Frank Stam, Victor Gerdes, Roel Hoogma, Martin Diekman, Martin Gerding, Cees Rustemeijer, Bernadette de Bakker, Joost Hoekstra, Aeilko Zwinderman, Jacques Bergman, Frits Holleman, Lorenzo Piemonti, Willem De Vos, Bart Roep, Max Nieuwdorp

**Affiliations:** 1 Department of Vascular Medicine, Amsterdam University Medical Centres, Amsterdam, Noord-Holland, The Netherlands; 2 Department of Internal Medicine, LUMC, Leiden, Zuid-Holland, The Netherlands; 3 Diabetes Research Institute, IRCCS San Raffaele Scientific Institute, Milan, Italy; 4 Diabetes Research Institute, San Raffaele Scientific Institute, Milan, Italy; 5 Internal Medicine, OLVG Location West, Amsterdam, North Holland, The Netherlands; 6 Internal Medicine, Rijnstate, Arnhem, Gelderland, The Netherlands; 7 Internal Medicine, OLVG, Location Oost, Amsterdam, Noord-Holland, The Netherlands; 8 Internal Medicine, North West Hospital Group, Alkmaar, Noord-Holland, The Netherlands; 9 Internal Medicine, Groene Hart Hospital, Gouda, Zuid-Holland, The Netherlands; 10 Internal Medicine, Deventer Hospital, Deventer, Overijssel, The Netherlands; 11 Internal Medicine, Hospital Amstelland, Amstelveen, North Holland, The Netherlands; 12 Department of Epidemiology and Biostatistics, Amsterdam University Medical Centres, Amsterdam, Noord-Holland, The Netherlands; 13 Department of Gastroenterology, Academic Medical Center, Amsterdam, The Netherlands; 14 Microbiology, WUR, Wageningen, The Netherlands; 15 Department of Diabetes Immunology, Diabetes & Metabolism Research Institute at the Beckman Research Institute, City of Hope, Duarte, CA, USA

**Keywords:** diabetes mellitus

## Abstract

**Objective:**

Type 1 diabetes (T1D) is characterised by islet autoimmunity and beta cell destruction. A gut microbiota–immunological interplay is involved in the pathophysiology of T1D. We studied microbiota-mediated effects on disease progression in patients with type 1 diabetes using faecal microbiota transplantation (FMT).

**Design:**

Patients with recent-onset (<6 weeks) T1D (18–30 years of age) were randomised into two groups to receive three autologous or allogenic (healthy donor) FMTs over a period of 4 months. Our primary endpoint was preservation of stimulated C peptide release assessed by mixed-meal tests during 12 months. Secondary outcome parameters were changes in glycaemic control, fasting plasma metabolites, T cell autoimmunity, small intestinal gene expression profile and intestinal microbiota composition.

**Results:**

Stimulated C peptide levels were significantly preserved in the autologous FMT group (n=10 subjects) compared with healthy donor FMT group (n=10 subjects) at 12 months. Small intestinal *Prevotella* was inversely related to residual beta cell function (r=−0.55, p=0.02), whereas plasma metabolites 1-arachidonoyl-GPC and 1-myristoyl-2-arachidonoyl-GPC levels linearly correlated with residual beta cell preservation (rho=0.56, p=0.01 and rho=0.46, p=0.042, respectively). Finally, baseline CD4 +CXCR3+T cell counts, levels of small intestinal *Desulfovibrio piger* and CCL22 and CCL5 gene expression in duodenal biopsies predicted preserved beta cell function following FMT irrespective of donor characteristics.

**Conclusion:**

FMT halts decline in endogenous insulin production in recently diagnosed patients with T1D in 12 months after disease onset. Several microbiota-derived plasma metabolites and bacterial strains were linked to preserved residual beta cell function. This study provides insight into the role of the intestinal gut microbiome in T1D.

**Trial registration number:**

NTR3697.

**Significance of this study**

**What is already known on this subject?**

- Gut microbiota are involved in human metabolic and autoimmune disease.
- Changes in faecal microbiota are associated with human type 1 diabetes (T1D).
- Animal studies have suggested that faecal transplantation can alter T1D.

**What are the new findings?**

- Faecal microbiota transplantation (FMT) stabilises residual beta cell function in subjects with new-onset T1D.
- These differential changes are accompanied by alterations in plasma metabolites, T cell autoimmunity, small intestinal gene expression as well as small intestinal and faecal microbiota composition.
- New correlations between changes in microbiota strains and plasma (targeted) metabolites in relation to small intestinal gene expression and T cell autoimmunity in human T1D were observed.

**How might it impact on clinical practice in the foreseeable future?**

- This study helps to quantify magnitude of gut microbiota-driven effects in humans with new-onset T1D using FMT.
- This study provides sample sizes for future trials and underscores that gut microbiota play a role in beta cell destruction seen in T1D subjects.

## Introduction

Type 1 diabetes mellitus (T1D) is an autoimmune disease characterised by progressive beta cell destruction. The T cell mediated autoimmune origin of T1D has prompted efforts to prevent disease progression by targeting T lymphocytes using immunosuppressive drugs including cyclosporine,^1^ anti-CD3 antibody treatment,^2^ antithymocyte globulin^3^ and anti-CD80 and anti-CD86 antibody treatment.^4 5^ However, these treatment strategies have (at best) a temporary impact on disease progression with no effect on long-term progression and are accompanied by serious side effects.^6 7^ Therefore, additional insights into T1D pathophysiology are urgently needed to find novel therapeutic interventions.

**Figure 1:**
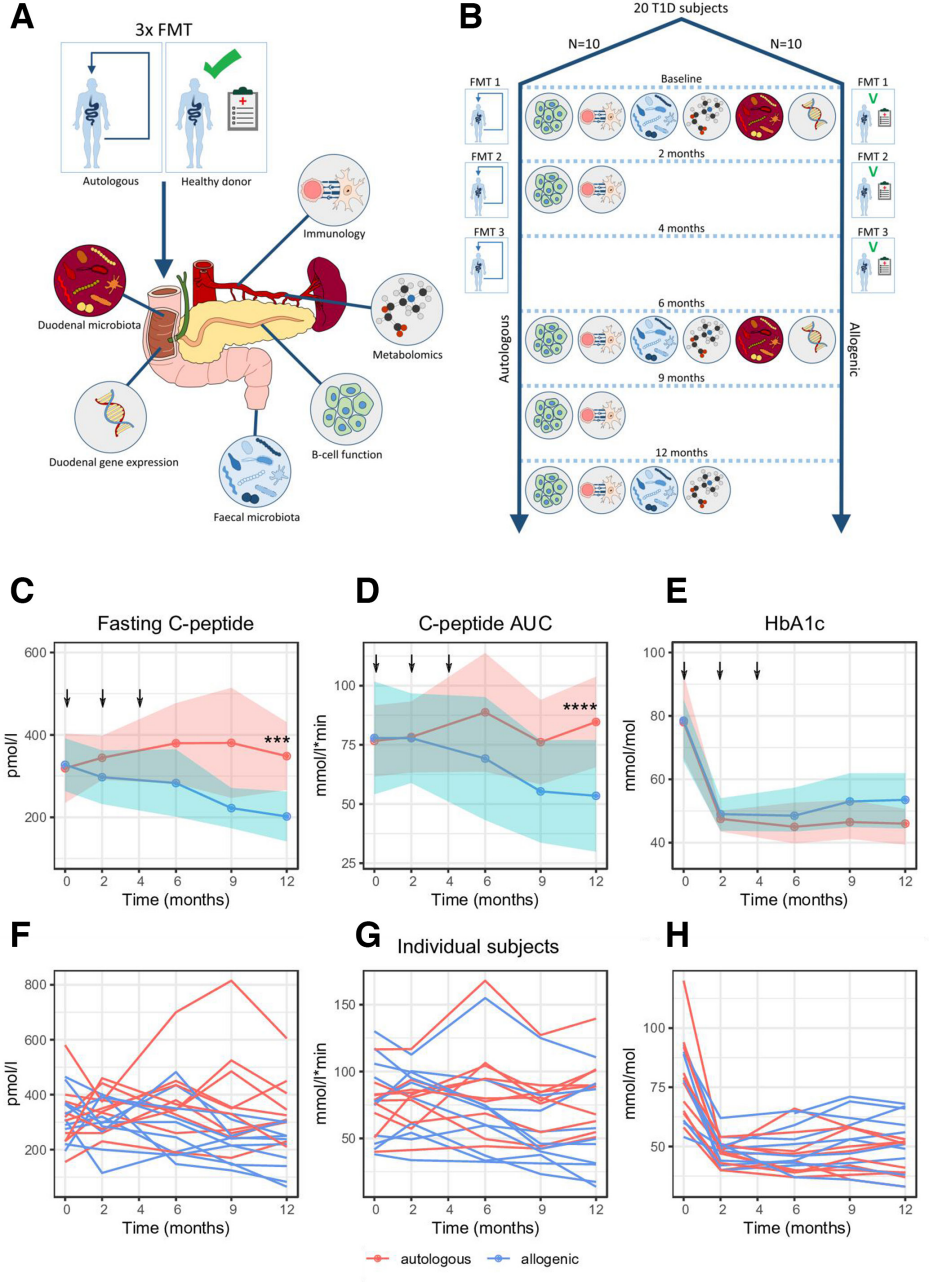
Schematic overview of study. (A) Study schematic showing which analyses were performed. (B) Study timeline showing FMTs were performed at 0, 2 and 4 months and which analyses where performed at each follow-up time point. (C) Change in fasting C peptide over time. Arrows indicate when FMT (allogenic: blue and autologous: pink with width of colour band indicating SD) was performed. Ribbons indicate CIs. Significance was calculated using LMM (see methods), *** p=0.00019. P values calculated using a Student’s t-test between groups at each time point were p=0.028 at 9 months and p=0.0049 at 12 months. (D) Change in C peptide AUC over time. Significance was calculated using LMM, **** p=0.000067. P value calculated using a Student’s t-test between groups at 12 months was p=0.033. (E) Change in A1c over time. Significance was calculated using LMM, p=0.12. P value calculated using a Mann-Whitney U test between groups at 12 months was p=0.19. SDs are depicted by the coloured width in the respective figures. (F, G and H) Individual trend lines for fasting C peptide, C peptide AUC and A1c respectively. AUC, area under the curve; FMT, faecal microbiota transplantation; LMM, linear mixed model; T1D, type 1 diabetes.

T1D pathophysiology has been linked to altered intestinal microbiota.^8–12^ Studies in non-obese diabetic (NOD) mice suggested that interaction of the intestinal microbes with the innate immune system is a critical factor for the development of T1D^13^ and can be improved by faecal microbiota transplantation (FMT) and specific microbes.^14^ Moreover, a growing number of studies point towards a role for the small intestinal immune system. For instance, in NOD mice segmented filamentous bacterial strains induce autoimmune diabetes by interaction with T-helper type 17 cells in the small intestinal lamina propria.^15^ Accordingly, infusion of bacterial strains into the pancreatic ductal system of a rat could induce T1D with pancreatic histological findings that mimic those observed in patients with T1D.^16^ Also, a recent study showed marked differences in small intestinal microbiota and duodenal gene expression between (longstanding) human T1D and healthy control subjects.^17^ T1D is thus believed to develop due to an altered intestinal epithelial barrier function induced by an impaired intestinal short-chain fatty acid (SCFA) production.^18^ This barrier is presumably necessary to prevent priming of the immune system to beta cell epitopes that are mimicked by harmful bacteria^19^ to which tolerance may be lost.^20^ Indeed, intestinal SCFAs butyrate and acetate administration were shown to improve beta cell function in NOD mice.^21^ However, we recently conducted a human intervention study in which butyrate administration had little immunological or metabolic effects in in T1D subjects.^22^ Finally, FMT is shown to be safe, can significantly alter the recipient gut microbiota composition (increasing butyrate producing bacterial strains) and can affect glycaemic control in metabolic syndrome subjects based on baseline microbiota.^23–25^ Therefore, this exploratory randomised controlled FMT trial in recent onset T1D subjects aimed to study the effects of sequential treatments of either healthy donor (allogenic) FMT or own (autologous) FMT on residual beta cell function (mixed meal test (MMT) stimulated C peptide response) during active FMT treatment (0–6 months) as well as long-term effects (0–12 months). Moreover, the relation with changes in duodenal microbiota composition, duodenal gene expression, faecal microbiota phylogenetic and metagenomic composition, whole blood T cell autoimmunity and fasting plasma metabolites was studied in these new-onset adult patients with T1D. A graphical summary of the study design is provided in figure 1A and B.

## Materials and methods

### Patient recruitment

New-onset patients with T1D were recruited from outpatient clinics in the Amsterdam region. Subjects aged 18–35 years with normal body mass index (BMI) (18.5–25 kg/m² and anti-GAD/IA-2 positive) were enrolled when diagnosed with T1D and with a maximum period of 6 weeks before inclusion and when there was still a residual beta cell function (plasma C peptide >0.2 mmol/L and/or >1.2 ng/mL after MMT). Exclusion criteria were a diagnosis or symptoms of another autoimmune disease, compromised immunity, use of any systemic medication (barring insulin) and use of antibiotics or proton-pump inhibitors in the last 3 months.

### Faecal donor recruitment, randomisation and FMT procedures

Lean (BMI <25 kg/m^2^), omnivorous, healthy male and female Caucasians were recruited to serve as faecal donors. Selection criteria are described in the online supplemental methods. Subjects were allocated in a 1:1 fashion using computerised randomisation to receive three autologous or allogenic faecal transplantations by nasoduodenal tube using freshly produced faeces at 0, 2 and 4 months (figure 1B) from the same sex matched donor as previously described^24^ and detailed in the online supplemental methods. All patients and investigators were masked to treatment assignment.

10.1136/gutjnl-2020-322630.supp1 Supplementary data

### Analysis of primary and secondary endpoints

A detailed description of each study visit can be found in the online supplemental methods. Mixed-meal tests (for residual beta cell function), intestinal microbiota analyses and immunological assays including fluorescent-activated cell sorting, lymphocyte stimulation assays (LST) and human leucocyte antigen multimer analyses to enumerate CD8 T cell autoimmunity to islet autoantigens (CD8 Quantum dot (QDot)) were performed at 0, 2, 6, 9 and 12 months. Targeted plasma metabolites (Metabolon, Morrisville, North Carolina, USA) were measured at 0, 6 and 12 months. Gastroduodenoscopy with duodenal biopsies was performed at 0 and 6 months to assess small intestinal microbiota and perform quantitative reverse transcription PCR to assess duodenal gene expression (see online supplemental table 1). Biometric measurements and glycaemic parameters were performed on all time points (figure 1B). For a detailed description of these analysis techniques, please refer to the online supplemental methods.

10.1136/gutjnl-2020-322630.supp2 Supplementary data

### Power calculation

A sample size of 17 patients in each group (34 patients in total) was needed to provide 80% power to detect a 50% difference in the Mixed Meal Test (MMT)-stimulated C peptide area under the curve (AUC) (360 mmol/L/min vs 180 mmol/L/min with an SD of 170 mmol/L/min) between treatment groups at 12 months^26 27^ with a two-sided test at α=0.05 and assuming a 10% dropout. This cut-off point was chosen because it is an established cut-off point in T1D research commonly employed by other intervention studies in T1D.^27^ All analyses were based on the prespecified intention-to-treat cohorts. Complete case analysis was done for the primary endpoint, the immunological parameters that are mentioned in the text and figures and for faecal microbiota and metabolites. Missing values in other (secondary) endpoints were assumed to be missing at random or completely at random. Details on missing values are found in the online supplemental methods (under subheading ‘missing values’). The primary endpoint of the trial was the preservation of (MMT stimulated) C peptide release at 6 and 12 months compared with baseline (0 months). This primary endpoint was thus chosen because this study focuses mainly on gut microbiota mediated effects on beta cell function. Although there are better clinical markers to monitor diabetes treatment effect such as A1c, homeostatic model assessment (HOMA) or number of daily insulin units, these are affected by endogenous insulin production and by diet, insulin compliance and insulin resistance; therefore, we did not consider these markers useful as primary endpoint for our study. The study was conducted at the Academic Medical Center (Amsterdam), in accordance with the Declaration of Helsinki (updated version 2013). All participants provided written informed consent. The study was prospectively registered at the Dutch trial registry (https://www.trialregister.nl/trial/3542). The safety of the patients was guarded by an independent Data Safety Monitoring Board (DSMB). Patients were not involved in the research process.

### Statistical analysis, machine learning and follow-up statistical analyses

Details regarding statistical analysis of the primary and secondary endpoints are described in the online supplemental methods. To identify which parameters (either as values at baseline or as relative changes) best predicted treatment groups and responders versus non-responders, we applied the Extreme Gradient Boosting (XGBoost) machine learning classification algorithm,^28^ in combination with a stability selection procedure.^29^ An overview of these predictive model analyses with area under the receiver-operator curve (AUROC) values and top three predictive features from each model is provided in online supplemental table S2. Details regarding these analyses are described in the online supplemental methods.

10.1136/gutjnl-2020-322630.supp3 Supplementary data

### Analysis of responders and non-responders irrespective of treatment group

Effects of autologous FMT were not surprising, as it affects homeostasis by introducing faecal microecology into the much less densely populated small intestine.^30^ Therefore, post hoc analyses were performed studying responders compared with non-responders to FMT, irrespective of treatment group, of which the most relevant features are shown in online supplemental table 2. Details regarding these analyses are described in the online supplemental methods.

## Results

Patients were included between 2013 and 2017. Patients with new-onset T1D (referred by their treating physician) were randomly assigned to donor FMT (n=11 subjects) or autologous FMT (n=10 subjects). One participant retracted consent after the first study visit before FMT intervention was performed. Due to lack of funding, the trial was stopped after 20 subjects were enrolled and completed the study protocol. Baseline characteristics are shown in table 1. Seven healthy lean donors (of whom three were used twice) donated for the allogenic gut microbiota transfer to patients with new-onset T1D, and the same donor was used for the three consecutive FMTs in an individual patient with T1D. There were no differences at baseline between both groups, and gastroenterological interventions were well tolerated in all subjects throughout the follow-up period. Also, there were no serious adverse clinical events nor adverse changes in plasma biochemistry observed.

**Table 1:** Baseline characteristics

Variable	Measure	Autologous group (n=10)	Allogenic group (n=10)	P value
Sex (M:F)	Amount	8:2	8:2	0.92
Age (at diagnosis, years)	Mean	25.0±3.5	24.3±5.4	0.73
Weight (kg)	Mean	75.0±13.0	71.0±10.9	0.46
BMI (kg/m²)	Mean	23.0±2.0	21.8±2.5	0.24
Insulin use per day (IU)	Mean	37±13	30±15	0.26
Daily insulin use (IU/kg/day)	Mean	0.49±0.13	0.43±0.24	0.55
HbA1c (mmol/mol)	Median	78 (66–90)	78.5 (67–90)	0.68
Fasting C peptide (pmol/L)	Mean	319±118	327±89	0.86
Microalbumin/creat ratio (mg/mmol)	Median	0.38 (0.34–0.41)	0.84 (-0.59–2.26)	0.31
C peptide AUC (mmol/L/min)	Mean	77±21	78±33	0.92
Anti-GAD (U/mL)	Median	110 (46–173)	103 (57–149)	0.85
Anti-IA2 (U/mL)	Median	696 (291–1094)	623 (345–901)	0.87
Ketoacidosis (DKA) at diagnosis	Amount	4/10	4/10	0.92
CRP (mg/L)	Median	0.8 (-20–22)	0.7 (-13–15)	0.83
Leukocytes (×10^9^/L)	Mean	5.7±2.5	6.0±1.3	0.71
Faecal calprotectin (mg/kg)	Median	42 (21–63)	26 (12–40)	0.15
Total cholesterol (mmol/L)	Mean	4.7±1.0	4.4±0.37	0.39
HDL-c (mmol/L)	Mean	1.41±0.31	1.58±0.43	0.34
LDL-c (mmol/L)	Mean	2.90±0.89	2.46±0.37	0.16
Triglycerides (mmol/L)	Mean	0.86±0.47	0.81±0.41	0.78
Total caloric intake (kcal/day)	Mean	1999±548	2051±512	0.83
Fat intake (g/day)	Mean	78±23	123±74	0.09
Sat. fat intake (g/day)	Mean	45±55	64±70	0.51
Protein intake (g/day)	Mean	124±73	99±35	0.34
Carbohydrate intake (g/day)	Mean	176±92	220±143	0.41
Fibre intake (g/day)	Mean	27±12	27±8	0.97

For normally distributed parameters, the mean is shown ±SD, and p values were calculated using a Student’s t-test and for not normally distributed parameters, the median with IQR (P25–P75) is shown, and the p value was calculated using Mann-Whitney U test.

Anti-GAD, antiglutamic acid decarboxylase; anti-IA2, anti-islet antigen 2; AUC, area under the curve; BMI, body mass index; CRP, C-reactive protein; DKA, diabetic ketoacidosis; HbA1c, hemoglobin A1c; HDL, high-density protein cholesterol; LDL, low-density protein cholesterol.

### Autologous FMT preserves (stimulated) C peptide levels compared with allogenic FMT

Mean fasting plasma C peptide at baseline was similar between groups (319 pmol/L±118 (SD) in the autologous group vs 327±89 in the allogenic group; p=0.86, Student’s t-test) but preserved in the autologous FMT group compared with deterioration the allogenic FMT group at 12 months (348 pmol/L±115 vs 202±85, Student’s t-test p value=0.0049; linear mixed models (LMMs) p=0.00019, figure 1C and F). A similar effect was seen in residual beta cell function as expressed by stimulated C peptide response AUC, which was equal at baseline (77 mmol/L/min±21 in the autologous group vs 78±33 in the allogenic group; p=0.92, Student’s t-test) but significantly more preserved at 12 months after autologous FMT (85 mmol/L/min±27 vs 53±33, Student’s t-test p value=p=0.033, LMM p value=0.000067, figure 1D and G). As expected, after exogenous insulin treatment started after T1D diagnosis A1c levels decreased in both the autologous and allogenic FMT groups at 12 months. Similar amounts of daily exogenous insulin (0.47 IU/kg/day vs 0.45 IU/kg/day, p value 0.71, respectively) were provided. No significant improvement of glycaemic control was noticed in the autologous FMT group compared with the allogenic FMT group (A1c 46 vs 53.5 mmol/mol, p=0.19, Mann-Whitney U test (MWU) p=0.19, LMM p value=0.12, figure 1E and H). Glucometabolic parameters at 0, 6 and 12 months are shown in table 2. Finally, weight, faecal calprotectin, microalbuminuria, lipid profiles and dietary intake (separate assessment of total calories, fat, saturated fat, protein, carbohydrates and fibre) were not different at baseline (table 1) nor during the course of the study (online supplemental figure S1A-E shows dietary parameters and S1F shows weight).

10.1136/gutjnl-2020-322630.supp4 Supplementary data

**Table 2:** 

Test	Baseline			6 months			12 months		
	Auto (n=10)	Allo (n=10)	P value	Auto (n=10)	Allo (n=10)	P value	Auto (n=10)	Allo (n=10)	P value
C peptide, fasting (pmol/L)	319±118	327±89	0.86	380±136	283±114	0.1	348±115	202±85	0.0045
C peptide, peak (t=90 min)(pmol/L)	766±264	748±369	0.9	855±350	671±371	0.27	805±255	511±342	0.043
C peptide, AUC (mmol/L/min)	77±21	78±33	0.92	89±35	69±36	0.24	85±27	53±33	0.032
Insulin dose (IU/kg/day)	0.49±0.13	0.43±0.24	0.55	0.41±0.10	0.37±0.18	0.57	0.47±0.10	0.45±0.18	0.71
HbA1c (mmol/mol)	78 (66–90)	78.5 (67–90)	0.68	45 (41–49)	48.5 (41–56)	0.41	46 (40–53)	53.5 (44–63)	0.19

The means±SD in the autologous and allogenic group at baseline, 6 and 12 months follow-up are shown. P values were calculated using the Student’s t-test. For HbA1c, the median and IQR (P25-P75) is shown, and the p value was calculated using a Mann-Whitney U test as it is not normally distributed. C peptide peak was measured at 90 min after ingestion of a mixed meal test. C peptide AUC designates the AUC of 120 min after the mixed meal with blood sampling at 0, 15, 30, 45, 60, 90 and 120 min.

AUC, area under the curve.

### T cell immunology changes in a similar fashion in autologous and allogenic FMT-treated group

A wide range of innate and adaptive immune cell phenotypes samples were analysed from whole blood (baseline medians in each group are listed in online supplemental table S3). Individual T cell responses against IA-2, GAD65 and preproinsulin (proliferation assay and LST) or blood frequencies of islet autoreactive CD8+ T cells (Qdot) showed no significant differential change between treatment groups using predictive modelling or MWU at the study time points 6 and 12 months. Similarly, frequencies of islet autoreactive CD8+ T cells did not differ significantly between treatment groups. In addition, FMT did not cause significant changes in the frequency of 35 leucocyte subsets as defined by flow cytometry (online supplemental figure 2). Of note, however, CD4+ CXCR3+ cells did change differentially between groups (p=0.01, MWU). The change between the baseline and 12 months correlated negatively with a change in our primary endpoint C peptide AUC (p=0.046, rho=−0.47)(online supplemental figure 3A-C). CD8+ CXCR3+ cells were different between study groups at baseline (p=0.0076, MWU). Change in CD8+ CXCR3+ cells also differed between treatment groups; however, this did not correlate with changes in C peptide AUC (online supplemental figure 3D-F).

10.1136/gutjnl-2020-322630.supp5 Supplementary data

### Treatment allocation of FMT is associated with changes in (small) intestinal gut microbiota composition and plasma metabolites

**Figure 2:**
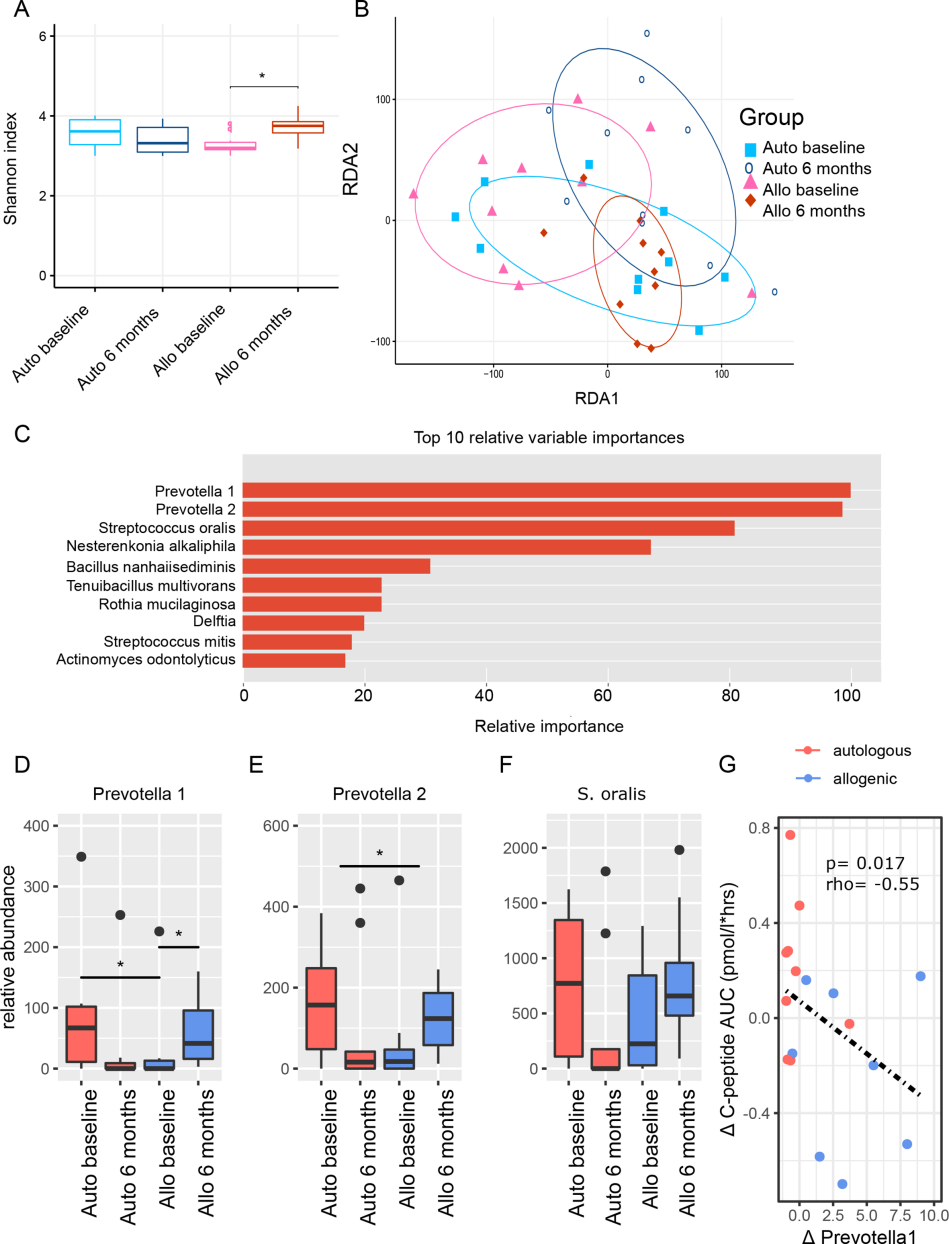
Small intestinal microbiota. (A) Boxplots of Shannon diversity between treatment groups at baseline and 6 months, which is the moment at which follow-up duodenal biopsies were taken. (B) RDA-plot showing clustering of treatment groups at baseline and at 6 months follow-up. (C) Top 10 small intestinal microbiota with relative importance that best predicted treatment group allocation allocation (XGBoost predictive modelling algorithm). Percentages are scaled towards the largest which is set at 100%. The top four microbiota stand out with higher relative importance. (D–F) Boxplots of top three small intestinal microbiota before and 6 months after FMT. P values were calculated using Mann-Whitney U test. The upper p value ‘(delta)’ was calculated by doing Mann-Whitney U test between the relative delta’s ((value after – value before)/value before) between treatment groups. Panel D: *Prevotella 1* auto baseline versus allo baseline p value=0.033, *Prevotella 1* allo baseline versus allo 6 months p value=0.049, *Prevotella 2* delta auto versus delta allo p value=0.048, *Streptococcus oralis* auto baseline versus auto 6 months p value=0.012. Figure part G shows the Spearman correlation between our top microbe *Prevotella 1* and our primary endpoint of Mixed Meal Test (MMT) stimulated C peptide release. FMT, faecal microbiota transplantation; RDA, redundancy analysis.

Alpha diversity of the small intestinal microbiota was not significantly different between treatment groups at baseline. At 6 months, there was a borderline significant difference between autologous and allogenic FMT group (p=0.054) concomitant with a significant increase in diversity in the allogenic FMT group (p=0.009; figure 2A). When plotted along ordination axes in a redundancy analysis (RDA-plot), small intestinal microbiota compositions clustered differently at baseline between groups and also changed between treatment groups (figure 2B). FMT treatment group allocation could be predicted reliably by change in specific small intestinal bacterial strains (AUROC 0.89±0.18 (CI)) including two species of *Prevotella* and *Streptococcus oralis* (figure 2C). However, changes on the phylum, family, genus and species level showed no major shifts in small intestinal microbiota composition (online supplemental figure 4). Relative abundances of all these species decreased after autologous faecal transplantation, but increased after allogenic faecal transplantation (figure 2D–F). Of note, the relative abundance of *Prevotella 1* showed a baseline difference between groups (p=0.033). The delta was significantly different between groups for *Prevotella 2* (p=0.048) but not for *Prevotella 1* (p=0.069) or *S. oralis*. Furthermore, a significant inverse correlation was observed between *Prevotella 1* relative abundance and stimulated C peptide AUC (Spearman p=0.015, rho=−0.55, see figure 2G). Of note, change in duodenal gene expression (measured at 0 and 6 months) did not predict treatment group allocation reliably (AUROC of 0.61±0.22).

### Faecal microbiota changes upon FMT

**Figure 3:**
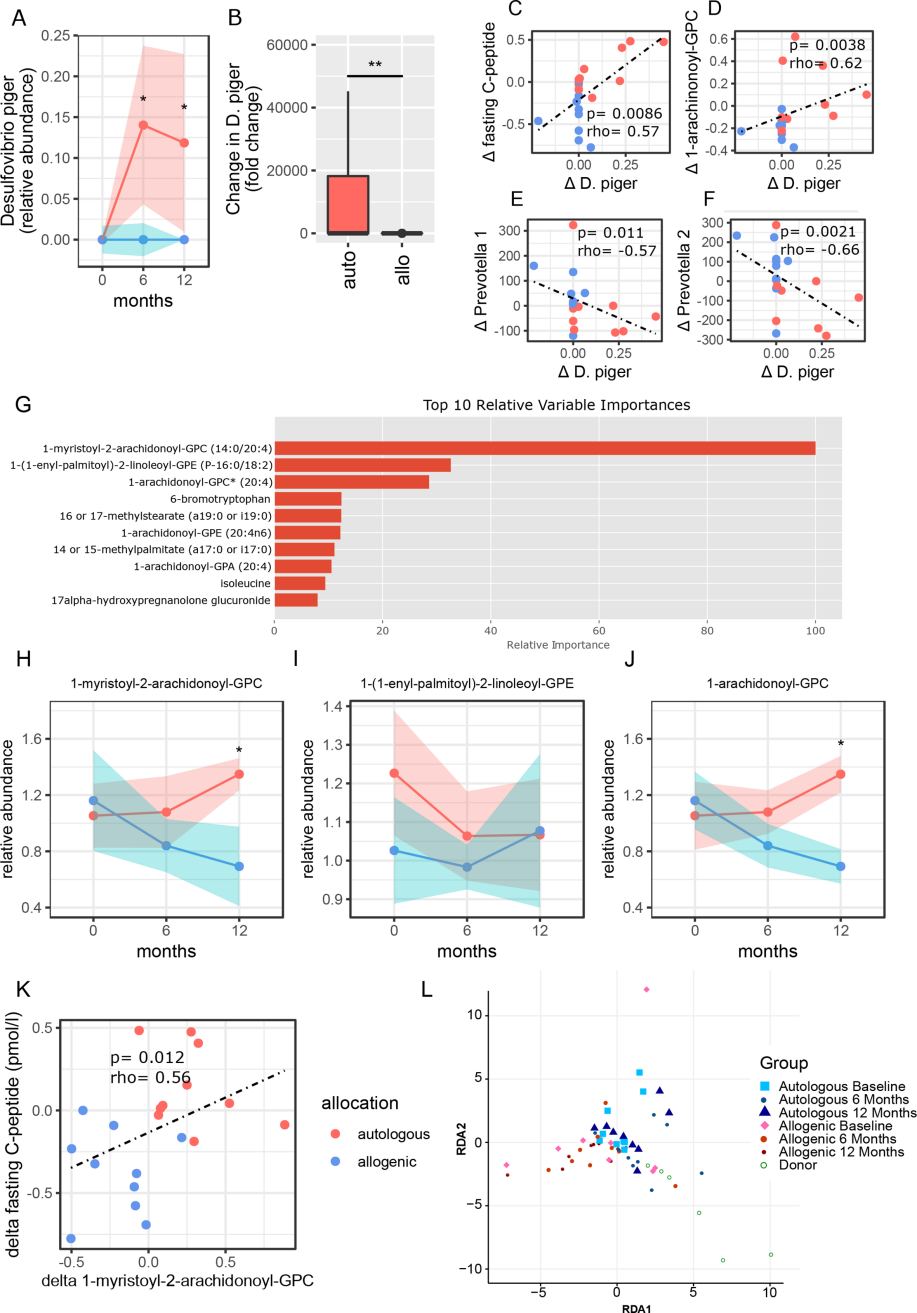
Correlations of clinical outcomes with plasma metabolites and *Desulfovibrio piger*. (A) Abundance of faecal *D. piger* over time (allogenic: blue, and autologous: pink with width of colour band indicating SD). P values were calculated using Mann-Whitney U test. At 6 months p value=0.024, at 12 months p value=0.023. (B) Fold change in *D. piger* between the groups (allogenic: blue and autologous: pink). The delta p value was calculated by doing Mann-Whitney U test on the delta’s between 0 and 12 months of each group, p value=0.006. (C) Spearman correlation plot of delta (0–12 months) faecal *D. piger* and delta (0–12 months) of fasting C peptide. (D) Correlation plot of faecal *D. piger* and 1-arachidonoyl-GPC. (E) Correlation plot of faecal *D. piger* and small intestinal *Prevotella 1.* (F) Correlation plot of faecal *D. piger* and small intestinal *Prevotella 2*. (G) Top 10 metabolites that best predicted treatment group allocation allocation (XGBoost predictive modelling algorithm). Percentages are scaled towards the largest, which is set at 100%. Top three metabolites stand out with higher relative importance in the analysis. (H–J) Relative abundance of top three metabolites plotted against time (allogenic: blue and autologous: pink with width of colour band indicating SD). Medians±IQR (P25–P75) are reported. P values were calculated using Mann-Whitney U test between groups at 12 months. 1-myristoyl-2-arachidonoyl-GPC is different between groups at 12 months, p value=0.020. 1-arachidonoyl-GPC is different between groups at 12 months, p value=0.020. (K) Spearman correlation between change in fasting C peptide and change in 1-myristoyl-2-arachidonoyl-GPC. (G) RDA of fasting plasma metabolites over time in T1D compared with healthy donors. T1D, type 1 diabetes.

Faecal microbiota composition was different between T1D and healthy donors at baseline and also changed differentially between treatment groups (online supplemental figure S5A and B). However, alpha diversity did not differ significantly between FMT treatment groups at baseline, 6 or 12 months nor between donors and recipients. Some shifts were seen on phylum, family, genus and species level between groups (online supplemental figure 5). Group allocation prediction based on faecal microbiota taxonomic changes between 0 and 12 months showed a moderate AUROC of 0.72±0.24. *Desulfovibrio piger* stood out as the most differentiating bacterial strain between treatment groups (online supplemental figure 6A). Treatment group prediction based on metabolic pathways showed a relatively poor AUROC of 0.68±0.27. The most differentiating metabolic pathway between both FMT groups was the seleno-amino acid biosynthesis pathway (online supplemental figure 6B). Interestingly, abundance of *D. piger* changed differentially between treatment groups at 6 (p=0.024, MWU) and 12 (p=0.023) months follow-up (figure 3A–B). Furthermore, change in *D. piger* correlated positively with change in fasting C peptide (p=0.009, figure 3C) and with plasma 1-arachidonoyl-GPC levels (p=0.004, figure 3D, this metabolite is discussed in the next paragraph). Moreover, a change in relative abundance of *D. piger* was inversely correlated with changes in relative abundance of both *Prevotella 1* (figure 3E) and *Prevotella* 2 (figure 3F).

### Plasma metabolite changes upon FMT

Treatment group allocation was predicted reliably by change in fasting plasma metabolites between 0 and 12 months (AUROC 0.79±0.23). The relative importance of the 10 most predictive metabolites are shown in figure 3G. From the top three metabolites, *1-myristoyl-2-arachidonoyl-GPC (MA-GPC*) (p=0.02, MWU) and *1-arachidonoyl-GPC* (A-GPC) (p=0.02), but not *1-(1-enyl-palmitoyl)−2-linoleoyl-GPE* (EPL-GPE), were different between groups at 12 months (figure 3H–J). Also, changes in plasma MA-GPC levels correlated significantly with changes in fasting C peptide (p=0.012, MWU, figure 3K) as well as overall plasma metabolites changes over time between FMT groups and donors (figure 3L).

### Baseline faecal microbiota composition, baseline faecal metabolic pathways and baseline duodenal gene expression predict FMT response

**Figure 4:**
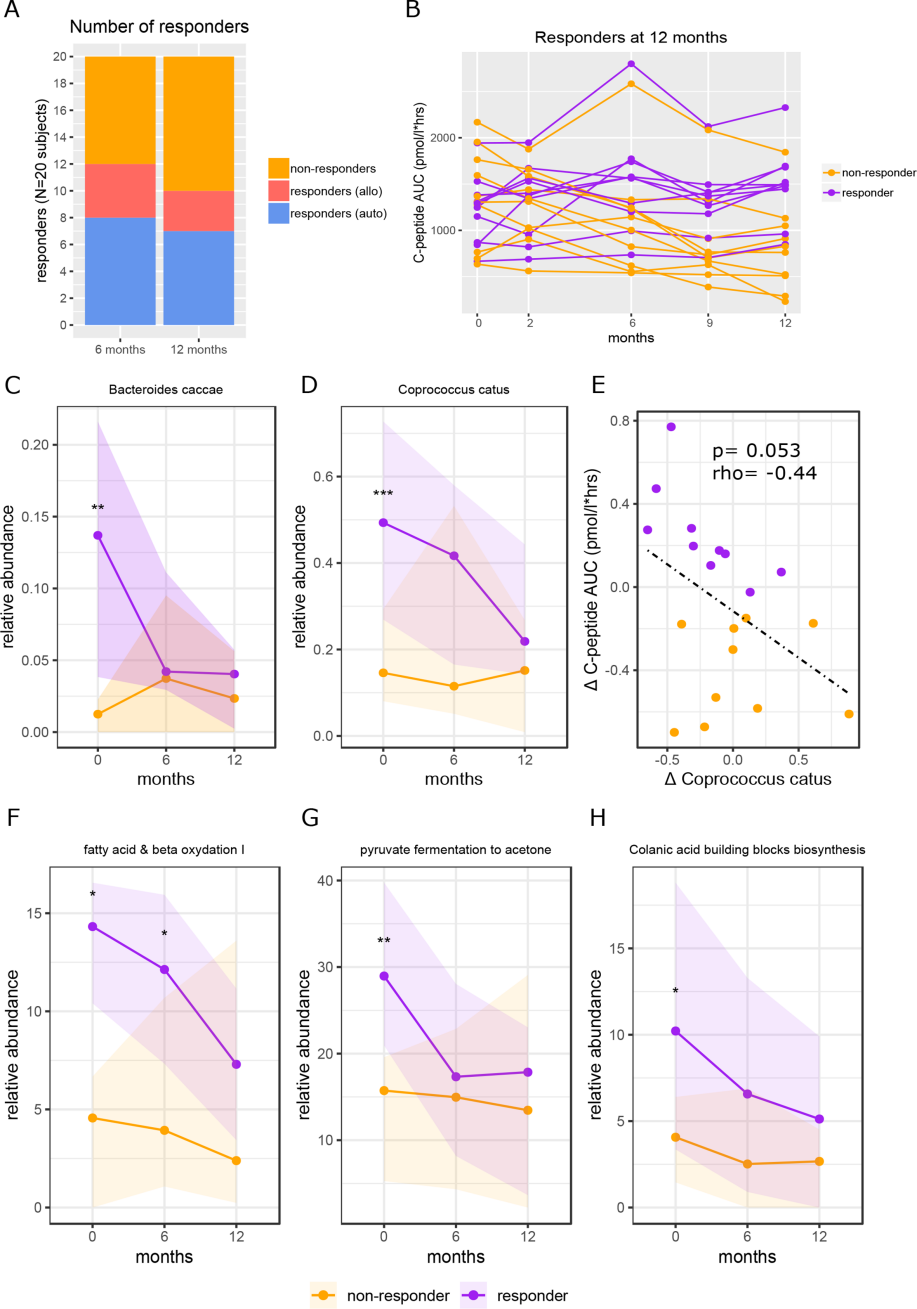
Baseline faecal microbiota and functional pathways in FMT clinical responders versus non-responders. Figure part A shows the number of responders at 6 months and at 12 months and how many subjects were in each treatment group. Response was defined as <10% decline in C peptide AUC compared with baseline. The 12 months responders were used for all analyses. Figure part B shows individual subject lines of C peptide AUC over time. Responders in purple and non-responders in yellow. Figure parts C and D show the abundance of *Bacteroides caccae* and *Coprococcus catus* over time, respectively. P values were calculated using Mann-Whitney U test between groups at each time point. For *B. caccae* at baseline the p value=0.0099, for *C. catus* at baseline the p value=0.00049. Figure part E shows the correlation between delta *C. catus* (0–12 months) and delta C peptide AUC (0–12 months). Spearman’s rho (r) is shown, and the p value was calculated using Spearman’s rank. Figure part F shows the relative abundance over time of fatty acid and beta oxidation, p value at baseline=0.014, p value at 6 months=0.011; figure part G shows the relative abundance over time of pyruvate fermentation to acetone, p value at baseline=0.0015; figure part H shows the relative abundance of time of the colonic acid building blocks biosynthesis pathways, p value at baseline=0.015. All p values were calculated using Mann-Whitney U test. AUC, area under the curve; FMT, faecal microbiota transplantation.

We next performed post hoc analyses to study if baseline faecal microbiota composition predicted clinical response on FMT (figure 4A–B), which indeed was the case (AUROC 0.93±0.14). In this regard, intestinal levels of *Bacteroides caccae* and *Coprococcus catus* stood out as most differentiating microbes (online supplemental figure 7), both of which were significantly more abundant at baseline in responders than in non-responders (figure 4C–D). Other differentiating intestinal bacterial strains, *Paraprevotella* spp, *Collinsella aerofaciens*, *Bacteroides eggerthii* and *Ruminococcus callidus* were also significantly different at baseline between responders and non-responders (online supplemental figure 8A-E). A borderline significant negative correlation was observed between change in *C. catus* abundance and stimulated C peptide AUC (p=0.053, r=−0.44, figure 4E).

In contrast, response to treatment was predicted less accurately by change in faecal microbiota composition (AUROC 0.76±0.23) than by baseline composition. Nevertheless, the species of which change best differentiated response were *Bacteroidales bacterium ph8, Actinomyces viscosus, Bacteroides thetaoitaomicron, Streptococcus salivarius, Ruminococcus bromii* and *Clostridium leptum* (online supplemental figure 9A), of which *B. bacterium ph8* (p=0.015, MWU) and *R. bromii* (p=0.013) became less abundant in responders versus non-responders, *S. salivarius* (p=0.045) became more abundant in responders versus non-responders and *B. thetaiotaomicron* was significantly different at baseline and showed a downwards trend in responders (online supplemental figure 9B-I).

Similarly, clinical response was more accurately predicted by baseline faecal microbial metabolic pathways (AUROC 0.85±0.22) than by change in faecal microbial metabolic pathways (AUROC 0.69±0.27). Metabolic pathways of which baseline abundance best predicted response included fatty acid and beta oxidation I, pyruvate fermentation to acetone and colanic acid building blocks biosynthesis (online supplemental figure 10), which were significantly higher in responders versus non-responders at baseline (p=0.014, p=0.0015 and p=0.015 respectively, MWU, figure 4F–H). However, there was no significant differential change in these pathways between responders versus non-responders. Also, neither baseline abundance of these pathways nor change in these pathways correlated with the primary endpoint (MMT stimulated C peptide response).

**Figure 5:**
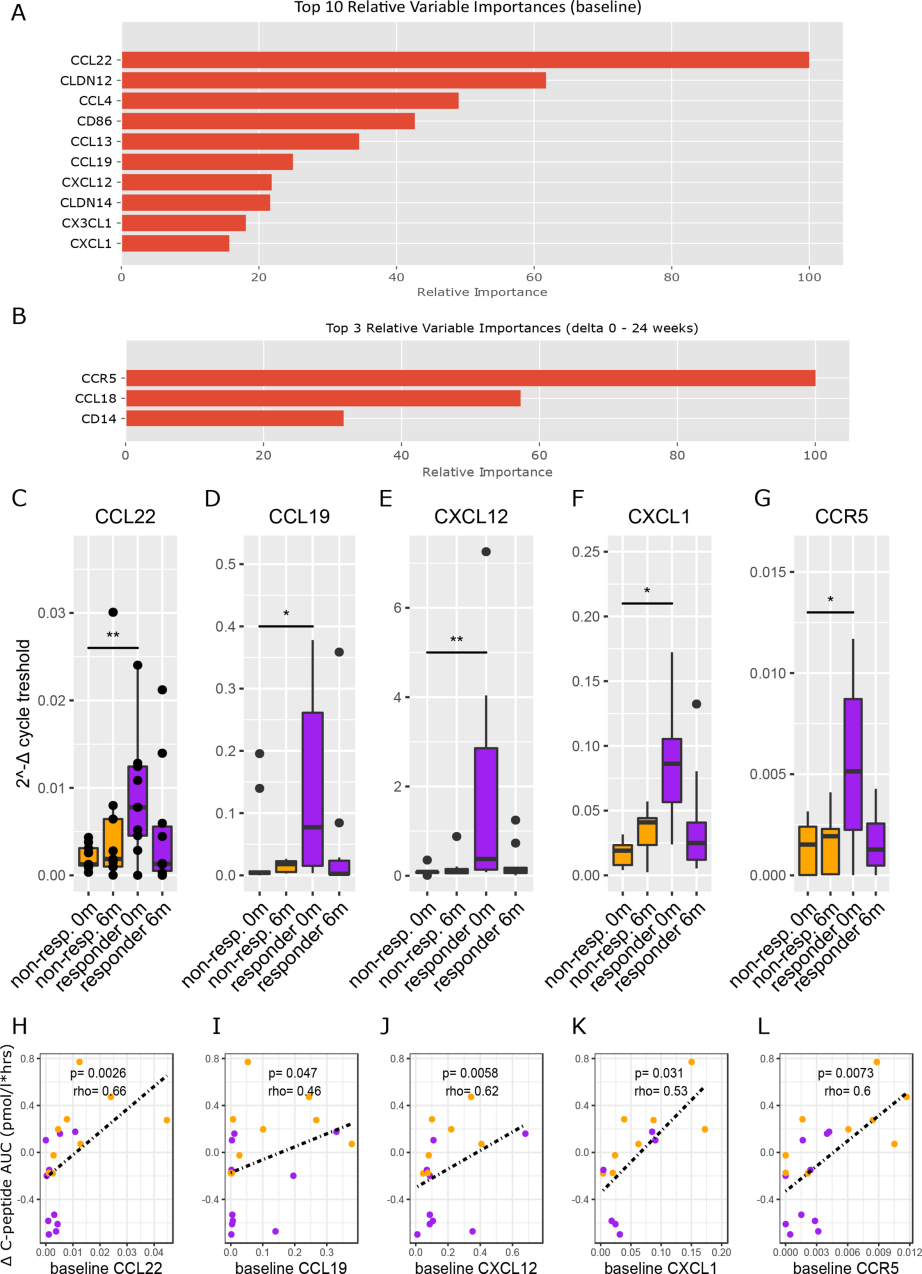
Duodenal gene expression in FMT clinical responders versus non-responders. Figure part A shows the top 10 genes of which baseline expression best differentiated responders from non-responders. Figure part B shows the top three genes of which change in gene expression (0–6 months) best differentiated responders from non-responders. Figure parts C–G show the genes from figure 5A that were significantly different between responders and non-responders at baseline. P values were calculated using Mann-Whitney U test between groups at each time point. Panel C p value=0.0039, panel D p value=0.011, panel E p value=0.0039, panel F p value=0.021, panel G p value=0.015. Figure parts H–L show the Spearman correlations between baseline expression of the genes from figure 5C–G and change in C peptide AUC. AUC, area under the curve; FMT, faecal microbiota transplantation.

In line, baseline duodenal gene expression predicted clinical response more accurately (AUROC 0.83±0.21) than change in duodenal gene expression (AUROC 0.73 ± 0.24). At baseline, the most differentiating genes were CCL22, CLDN12, CCL4, CD86, CCL13, CCL19, CXCL12, CLDN14, CX3CL1 and CXCL1 (figure 5A), while CCR5 and CCL18 (figure 5B) were the genes with the most notable differential change. Expression of several of these genes was significantly different between responders and non-responders at baseline: CCL22 (p=0.0039, MWU), CCL19 (p=0.011), CXCL12 (p=0.0039), CXCL1 (p=0.021) and CCR5 (p=0.015) (figure 5C–G). Moreover, baseline values of these genes correlated well with change in stimulated C peptide AUC (figure 5H–L). Interestingly, all these genes decreased after FMT treatment, but only the decrease in CCL19 (p=0.049) was statistically significant. Finally, gene expression of tight junction protein CLDN12 was high in non-responders at baseline (online supplemental figure S11A), while gene expression of CCL4 and CD86 were higher in responders (online supplemental figure S11B and C).

### Integration of multiomics analyses

**Figure 6:**
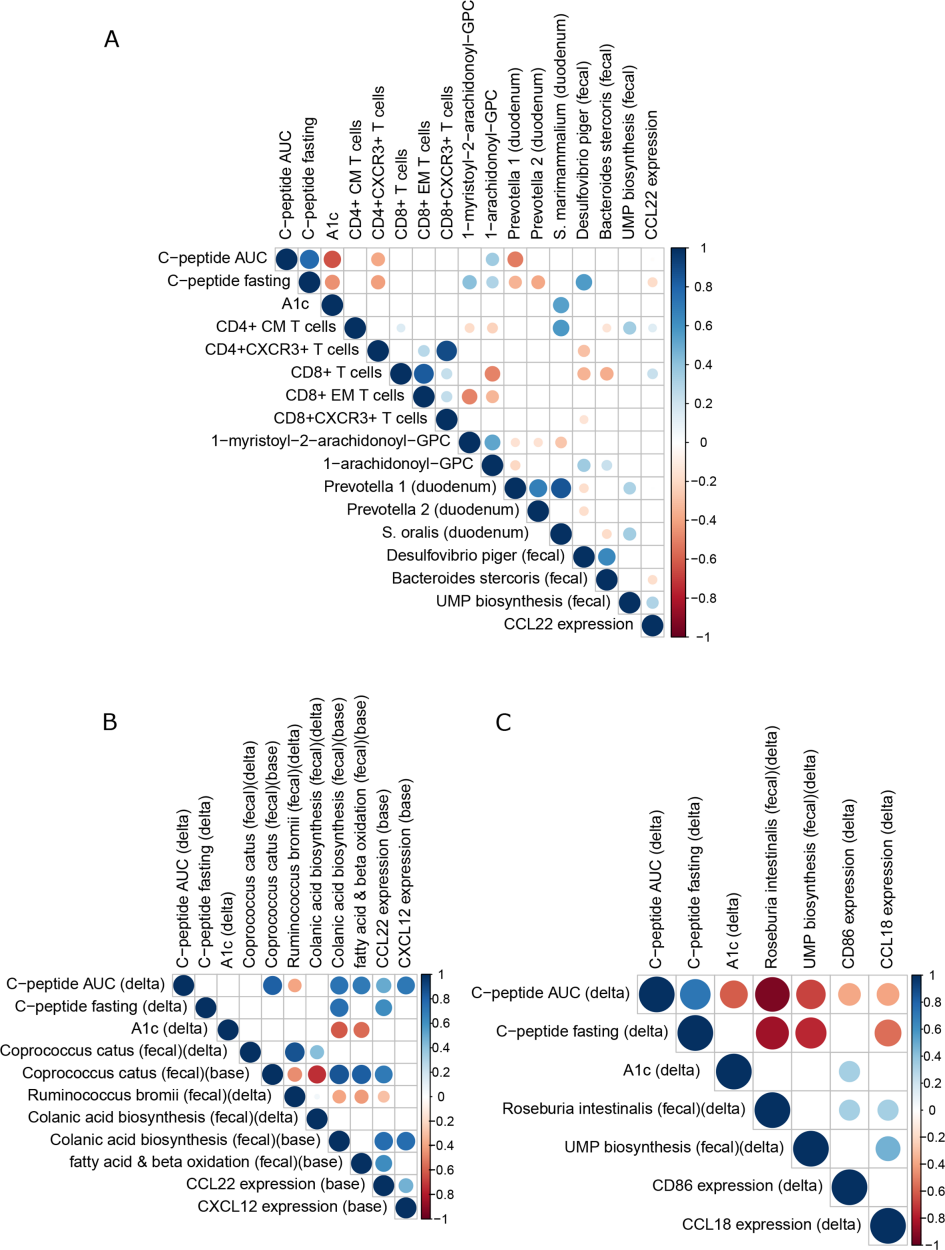
Correlation plots with altered plasma metabolites, bacterial strains and residual beta cell function on FMT. (A) Plot showing Spearman correlations of all subjects pooled (n=20). Only significant (p<0.05) correlations are shown. Red designates a negative correlation and blue a positive correlation. Dot size corresponds to p value (larger is smaller) and dot colour to correlation strength (Spearman’s rho). This plot was derived from a larger plot from which all parameters that did not correlate with our primary endpoint and/or any key parameters were removed. (B) As figure part A, for autologous treatment group. (C)aAs figure part A, for the allogenic treatment group. AUC, area under the curve; FMT, faecal microbiota transplantation.

Correlations between parameters found to be significantly affected by FMT were explored. Since responders were found in both treatment groups, correlations were first explored in our pooled dataset (n=20) (figure 6A) and then within treatment groups separately (figure 6B and C) and in clinical responders to FMT (online supplemental figure S12). In the pooled dataset (figure 6A), an intertwined cluster of notable parameters was found which positively and negatively associated with markers of glucose regulation (ie, C peptide AUC, fasting C peptide and A1c; figure 6A). On one hand, the highly correlated plasma metabolites MA-GPC and A-GPC accurately predicting preservation of insulin secretion, correlate positively to *D. piger*, which correlates positively to fasting C peptide. On the other hand, *Prevotella 1*, *Prevotella 2* and *S. oralis* correlate negatively to glucose regulation and to the metabolites MC-GPC and A-GPC. In addition, residual beta cell function correlates negatively to CCL22 activity and CD4+ CXCR3+ T cells, which in turn correlate negatively to *D. piger*. Analysing treatment groups separately, preserved beta cell function (high C peptide) in the autologous group was characterised at baseline by high *C. catus*, high induction of the colanic acid biosynthesis, fatty acid and beta oxidation pathways and high CCL22 and CXCL12 expression, as well as a subsequent decrease in *R. bromii*, which correlates negatively with these two pathways and CCL22 at baseline (figure 6B). In the allogenic group, preserved beta cell function was characterised by a decrease in faecal *Roseburia intestinalis* and a decrease in the UMP biosynthesis pathway (which incidentally correlates positively with *Prevotella 1* and *2*) and a decrease in CD86 and CCL18 expression, which were both higher in responders at baseline and subsequently decreased. Both CD86 and CCL18 genes in turn correlate with *R. intestinalis*, while CCL18 in addition correlates positively with the UMP biosynthesis pathway (figure 6C). Finally, in clinical responders, preserved beta cell function was characterised by decreases in duodenal *Prevotella 1*, *Prevotella 2*, faecal *C. catus*, metabolite EPL-GPE, the pathway fatty acid and beta oxidation and CD4+ CXCR3+ T cell s, whereas *D. piger* increased (online supplemental figure S12).

## Discussion

We here report for the first time that FMT can have an effect on residual beta cell function in new-onset T1D. This accords with recent observational studies supporting a role for the intestinal microbiota in T1D subjects.^8–12^ In contrast to our hypothesis, autologous FMT performed better than healthy donor FMT, while even in the allogenic group, the decline in MMT stimulated C peptide response appeared less than expected in T1D without treatment in 1 year.^26 27^ An appealing explanation would be that beneficial immunological effects of FMT (irrespective of donor source) are more pronounced and durable when the FMT donor microbiota is more immunologically compatible with the host. We suspect that allogenic FMT increases the already present increase in inflammation that is known to occur around the time of diagnosis,^31^ by offering immunologically foreign colonic microbiota to which the host is less tolerant to the small intestine (where the T cells are thought to be trained^32^), which may overshadow beneficial effects that occur simultaneously and are caused by different agents. In contrast to animal studies, the beneficial effect of FMT was not associated with changes in SCFA-producing strains.^21^ Nevertheless, observations point towards an immunological regulatory role of specific plasma metabolites that are derived from diet and converted by intestinal microbiota.^33^

### Preservation of beta cell function by autologous FMT is T cell mediated

A number of studies targeting T cells have shown delayed loss of beta cell function in T1D.^1 3–5 34 35^ Our study underscores that beta cell preservation after transplantation of host colonic microecology is T cell mediated, as CD4+ CXCR3+ and CD8+ CXCR3+ T cells were decreased differentially in the responders at 12 months. Beta cells are known to attract autoreactive T cells through the production of ligands (ie, CXCL9, 10 and 11) that bind to CXCR3.^36–38^ Also, it is known that the putative immunological changes occur not peripherally but locally in the pancreas and draining lymph nodes, the small intestinal mucosa or the gut-draining lymph nodes.^39^ Indeed, altering tone of the regulatory T cells residing in the small intestinal mucosa can prevent T1D.^40 41^ Furthermore, we identified that baseline expression of CCL22 in small intestine was a strong predictor of clinical response. It has been previously published that small intestinal CCL22 expression is higher in T1D subjects versus controls,^17^ and CCL22 has been previously suggested as novel therapeutic strategy for T1D, for example, protecting against autoimmunity in NOD mice by activating and recruiting regulatory T cells and decreasing the number of CD8+ T cells.^42 43^ CCL4 expression was also higher in our responders, while in NOD mice CCL4 is required in protection from T1D by neutralising IL-16^44^ and is also required by T cells for IL-4-mediated protection from T1D.^45^ Also, small CD86 expression was higher in our clinical responders than in non-responders, which is interesting as CD86 is required for full T cell activation and also a target of Abatacept, which can postpone decline beta cell function in T1D subjects.^4 46^

### Preservation of beta cell function is associated with changes in specific gut microbiota strains

In line with previous literature,^47^ we propose that *D. piger* dampens autoimmunity in T1D via *plasma 1-arachidonoyl-GPC* thus affecting CXCR3+ T cells. Predictive modelling showed that baseline faecal microbiota taxonomy and metabolic pathways accurately predicted response at 12 months. However, the identified microbes (eg, *B. caccae* and *C. catus*) did not correlate with any of our relevant immune parameters, small intestinal genes or plasma metabolites. This suggests that faecal microbiota composition is consequence rather than cause of the host immunological characteristics that associate with response. The exception to this was *D. piger*, a sulfate-reducing bacterial strain that was previously shown to shape individual responses of gut microbiota to diet.^48^ Its beneficial effects may be mediated by its production of hydrogen sulfide, a molecule that was found to have neurostimulatory effects^49^ and affect regulatory T cells and immune homeostasis.^50^ Moreover, we identified *D. piger* as outstanding faecal microbial predictor of FMT treatment group allocation. Interestingly, this small intestinal bacterial strain was also beneficially associated with change in stimulated C peptide responses on FMT and its abundance increased in the autologous group and in the overall responders. Interestingly, *D. piger* correlated positively with levels of plasma 1-arachidonoyl-GPC (figure 3I), one of our key metabolites that also associated with improved C peptide production. Moreover, *D. piger* and this metabolite correlate negatively with CD4+ CXCR3+ and CD8+ CXCR3+ T cells, which is in line with previous reports in murine T1D.^51^ In conclusion, *D. piger* could be a strong candidate to dampen autoimmunity by suppressing these cells through production of A-GPC, for example, through uptake by protruding dendrites of immune cells into the intestinal lumen.^52^ Interestingly, *D. piger* was recently cultured from the human intestinal tract, enabling testing this bacterial strain in human T1D.^53^ Other bacterial species in the duodenum that best differentiated between treatment groups were two unnamed *Prevotella* spp and S. *oralis*. In this regard, faecal^8^ but not duodenal *Prevotella* has been previously linked to T1D. Our explorative integration of multiomics analyses subsequently show that these *Prevotella* spp and *S. oralis* are negatively associated with our key beneficial metabolite MA-GPC, a glycerophospholipid. In this regard, other phospholipids have previously been linked to beta cell function in new-onset T1D.^26^ *B. stercoris* correlated positively with *D. piger* and A-GPC and negatively with *S. oralis* and CCL22, but did not correlate positively with C peptide. Intriguingly, *B. stercoris* was recently found to be cross-recognised by ZnT8-reactive CD8+ T cells.^19^ Finally, changes in *R. bromii* (autologous FMT group) and *R. intestinalis* (allogenic FMT group) were negatively associated with changes in C peptide, although both strains are generally regarded as beneficial microbes that thrive during fibre-rich diets, produce SCFAs and promote intestinal integrity.

### Limitations

First, this exploratory RCT stopped enrolment before the calculated sample size was reached. It is of limited sample size, and it was not powered for secondary clinical endpoints such as A1c. However, it paves the way for larger studies to confirm our findings. Although the driving factors of baseline gut microbiota composition for FMT treatment efficacy in new-onset T1D are currently unknown, we speculate that the level of clinical response might be driven by gut microbial strain composition in the FMT (irrespective of donor source) in combination with host factors such as autoimmunological tone. Whether adding a standard dietary intervention could work synergetic with FMT donors better matched to host immunology to optimise clinical metabolic and immunological response requires further study. Second, we attempted to approximate local effects of our intervention by taking duodenal mucosal biopsies at baseline and after 6 months (thus during the active FMT intervention). However, most relevant immunological effects are expected to occur in the pancreas and the pancreatic lymph nodes, compartments that cannot be sampled in living T1D patients. Third, our earliest biological samples were taken 2 months after first FMT. Therefore, changes that may have occurred sooner but have waned may have been missed. Fourth, our population consisted of only adult subjects with consequently slower onset T1D, which may be immunologically different from earlier onset adolescent T1D.^54^ Notwithstanding and awaiting confirmation of this pilot trial in a larger RCT with adult T1D patients, our study also warrants trials applying FMT in younger T1D subjects. Fifth, although insulin resistance plays a modest role in T1D, we have not quantified it in this study. As shown in previous research, insulin sensitivity can be both increased^23 24^ and decreased^25^ by donor FMT. However unlikely in a state of beta cell failure and absolute insulin deficiency, it is conceivable that FMT has increased insulin sensitivity thereby counteracting increased C peptide release and obscuring observable benefits. Finally, in future studies, we should include a true placebo control group (eg, lavage and duodenal tube placement without FMT) to compare autologous FMT infusions with the ‘natural’ course of beta cell function decline in new-onset T1D.

## Conclusions

Faecal transplantation of colon-derived microbiome into the host small and large intestine in patients with new onset T1D effectively prolongs residual beta cell function in our study. From this hypothesis-generating study, we report several important findings. First, several novel bacterial strains including faecal *D. piger* and *B. stercoris* as well as duodenal *Prevotella* spp and *S. oralis* were identified with therapeutic potential. Accordingly, increases in plasma phospholipids and tryptophan derivatives such as 1-myristoyl-2-arachidonoyl-GPC and 1-arachidonoyl-GPC as well as 6-bromotryptophan after FMT associated with beneficial changes in small intestinal CCL22 expression and whole blood immune cell subsets such as CXCR3+ CD4+ T cells. While developing the identified leads for assessment in clinical trials in T1D will be challenging and time consuming, FMT itself appears to be a safe treatment modality that can be readily applied in clinical studies to dissect the causal influences of gut microbiota in pathophysiology of T1D. We therefore hope that our exploratory study will spark larger randomised (allogenic vs autologous vs real placebo) FMT trials with a longer follow-up to confirm and expand on our compelling findings of FMT-based intervention in the progressive loss of beta cell function in human T1D.
