# Supplementary material for: Faecal microbiota transplantation halts progression of human new-onset type 1 diabetes in a randomised controlled trial
Source: Gut. 2020 Oct 26;70(1):92–105. doi: 10.1136/gutjnl-2020-322630 (PMC7788262; doi:10.1136/gutjnl-2020-322630)
Supplement: Supplementary data [file gutjnl-2020-322630supp001.pdf]

BMJ Publishing Group Limited (BMJ) disclaims all liability and responsibility arising from any reliance placed on this supplemental material which has been supplied by the author(s)

Supplementary methods

Abbreviations

ASV – amplicon sequence variant

AUC – area under the curve

AUROC – area under the receiver-operator curve

CMV - cytomegalovirus

CRP – C-reactive protein

EBV – Epstein-Barr virus

ESBL – extended-spectrum beta lactamase

FACS – fluorescent-activated cell sorting

FMT – fecal microbiota transplantation

GAD – glutamate decarboxylase

HDLc – high density lipoprotein cholesterol

HLA – human leukocyte antigen

LDLc – low density lipoprotein cholesterol

LMM – linear mixed models analysis

LST – lymphocyte stimulation test

MMT – mixed meal test

MWU – Mann-Whitney U test

MRSA **–** methicillin-resistant *Staphylococcus aureus*

PBMCs - Peripheral blood mononuclear cells

PCR – polymerase chain reaction

PPI – preproinsulin

Qdot – quantum dot

ROC – receiver-operator curve

RT qPCR – reverse transcription quantitative PCR

T1D – type 1 diabetes

TG - triglycerides

TT – tetanus toxoid

UPLC-MS/MS - ultra high performance liquid chromatography coupled to tandem mass spectrometry

Fecal donor recruitment and randomization

Fecal donors completed questionnaires regarding dietary and bowel habits, travel history, comorbidity including family history of diabetes mellitus and medication use. They were screened for the presence of infectious diseases as described previously[1]. Furthermore, donors with 1st or 2nd degree relatives with autoimmune diseases (including Coeliac disease, autoimmune thyroid disease, type 1 diabetes and rheumatoid arthritis) were excluded. Blood was screened for human immunodeficiency virus; human T-lymphotropic virus; Hepatitis A, B, and C; cytomegalovirus (CMV); Epstein–Barr virus (EBV); strongyloides; amoebiasis, and lues. Presence of infection resulted in exclusion, although previous and non-active infections with EBV and CMV were allowed. Donors were also excluded if screening of their feces revealed the presence of pathogenic parasites (e.g. blastocystis hominis, dientamoeba fragilis, giardia lamblia), multiresistent bacteria (*Shigella*, *Campylobacter*, *Yersinia*, *MRSA ,ESBL, Salmonella, enteropathogenic E. Coli* and *Clostridium difficile*) or viruses (noro-, rota-, astro-, adeno (40/41/52)-, entero-, parecho- and sapovirus) as previously recommended[2]. After an overnight fast, plasma samples were taken for biochemistry and metabolomics and a morning fecal sample was collected.

FMT procedure

Seven healthy lean donors (of whom 3 were used twice) donated for the allogenic gut microbiota transfer to new onset type 1 diabetes (T1D) patients, and the same donor was used for the three consecutive FMT’s in an individual T1D patient.

After admission, a duodenal tube was placed by gastroscopy or CORTRAK enteral access system. Each patient then underwent complete colon lavage with 2-4L of Klean prep® (macrogol) by duodenal tube until the researcher judged that the bowel was properly lavaged (i.e. no solid excrement, but clear fluid) for approximately 3h. Then, between 200 and 300 grams of feces was processed by dilution in 500 ml of 0.9% saline solution and filtered through unfolded cotton gauzes. The filtrate was used for transplantation two hours after the last administration of Klean prep® by duodenal tube in around 30 minutes using 50cc syringes. After a short observation period the patient was sent home.

Study visits

All study visits were performed at Amsterdam UMC, location AMC. Participants were asked to fill out an online nutritional diary for the duration of one week before each study visit to monitor caloric intake including the amount of dietary carbohydrates, fats, proteins and fibers. During the study visits blood pressure, weight and daily insulin use were documented. Fasting blood samples were taken at each visit and upon centrifugation stored at -80°C for subsequent analyses. Whole blood sodium heparin tubes were kept on room temperature and processed within 24 hours for immunological analyses (described under immunology).

Description per study visit

All visits took place after an overnight fast with subjects taking no long acting insulin the night before as previously described (Moran et al., 2013). At each visit blood, fecal and urine sampling and biometric measurements took place. At baseline all patients first underwent gastroduodenoscopy. A small dose of midazolam (2.5 or 5mg) was administered for patient's comfort. Duodenal biopsies were immediately collected in sterile tubes, snap-frozen in liquid nitrogen and stored at -80°C, followed by nasoduodenal tube placement. Then at least 2 hours later, a standardized 2h mixed meal test (MMT)(Nestlé sustacal boost®) was performed as previously described[3] to study residual Beta-

cell function. At 2, 9 and 12 months, patients again underwent a mixed-meal test for residual Beta-cell C-peptide secretion. After the 2 hour MMT, a duodenal tube was placed by means of CORTRAK enteral access, bowel cleansing for 6 hours was performed and the fecal transplant procedures were repeated. At 6 months, patients underwent gastroduodenoscopy and biopsies were taken from the duodenum and again thereafter, the mixed-meal test was performed. Of note, the similar daily schedule was used in all patients to minimize variation in measurements between subjects.

Mixed meal test

Starting the evening before each mixed meal test, T1D patients interrupted their long-acting insulin injections as previously published [3]. After an overnight fast and without taking their short-acting morning insulin dose, a mixed meal test was performed with Boost High Protein (Nestlé Nutrition, Vervey, Switzerland) at 6 ml/kg body weight with a maximum of 360 ml per person as previously described[4]. Subsequent blood sampling for stimulated C-peptide was performed at -10, 0, 15, 30, 45, 60, 90 and 120 minutes. Area under the curve (AUC) was derived according to the trapezoidal rule.

Adaptive T-cell Immunity

Whole blood samples were processed within 24 hours after sampling. Peripheral blood mononuclear cells (PBMC's) were used for measurement of immune response. Granulocytes were isolated for DNA-extraction and human leukocyte antigen (HLA) typing.

Isolation of Peripheral blood mononuclear cells (PBMC's)

PBMC's were isolated using Ficoll-density gradient centrifugation (ficoll 5.7%, amidotrizoaat 9%, *Pharmacy Leiden University Medical Centre*). After centrifuging, the interphase containing PBMC's was harvest and washed 3 times using PBS. PBMC's were suspended in 2 ml Iscove's modified

BMJ Publishing Group Limited (BMJ) disclaims all liability and responsibility arising from any reliance placed on this supplemental material which has been supplied by the author(s)

Dulbecco's Medium (IMDM, *Lonza*) supplemented with L-glutamine, penicillin-streptomycin (Pen Strep) and 15% Human serum and counted.

Lymphocyte Stimulation Test (LST)

T-cell proliferation in response to antigenic stimulation was performed as described previously (Kracht, Nature Medicine 2017). Cells were incubated in conditioned medium alone or in the presence of autoantigen proteins glutamate decarboxylase (GAD65), preproinsulin (PPI), insulinoma antigen-1 (IA-2) and a defective ribosomal product of proinsulin mRNA (DRiP) generated by stressed Beta cells[5]. For controls, cells were stimulated with Interleukin-2 (IL-2) or cultured with tetanus toxoid (TT). Cells were incubated for 5 days, after which ^{3}H-thymidine (50µl, 10 µCi/ml) was added for the last 18 hours of the culture.

Fluorescent-activated cell sorting (FACS) analyses and Quantum dot (Qdot)

For phenotyping and quantification of autoreactive CD8+ T-cell s, PBMC were stained with fluorescent antibodies according to a standard, independently validated protocol as described previously [6]. Stained cells were measured using FACS-Canto (phenotyping) and LSR-II (Q-dot) machines (Becton&Dickinson). Phenotyping data were analyzed using FlowJo software (TreeStar) using the gating strategy (supplementary figure 1) or as described previously for Qdot analyses [6].

Plasma metabolites

Fasting plasma targeted metabolite measurements were done by Metabolon (Durham, NC), using ultra high performance liquid chromatography coupled to tandem mass spectrometry (UPLC-MS/MS), as previously described [7]. Raw data was normalized to account for inter-day differences. Then, the levels of each metabolite were rescaled to set the median equal to 1 across all samples. Missing values, generally due to the sample measurement falling below the limit of detection, were then imputed with the minimum observed value for the respective metabolite.

BMJ Publishing Group Limited (BMJ) disclaims all liability and responsibility arising from any reliance placed on this supplemental material which has been supplied by the author(s)

Biochemistry

Glucose and C-reactive protein (CRP, Roche, Switzerland) were determined in fasted plasma samples. C-peptide was measured by radioimmunoassay (Millipore, Amsterdam, The Netherlands). Total cholesterol, high density lipoprotein cholesterol (HDLc), and triglycerides (TG) were determined in EDTA-containing plasma using commercially available enzymatic assays (Randox, Antrim, UK and DiaSys, Germany). All analyses were performed using a Selectra autoanalyzer (Sopachem, The Netherlands). Low density lipoprotein cholesterol (LDLc) was calculated using the Friedewald formula. Calprotectin was determined in feces using a commercial ELISA (Bühlmann, Switzerland). Hba1c was measured by HPLC (Tosoh G8, Tosoh Bioscience)

Fecal sample shotgun sequencing and metagenomic pipeline

Fecal microbiota were analysed using shotgun sequencing on donor and patient samples taken at 0, 6 and 12 months after initiation of study. DNA extraction from fecal samples for shotgun metagenomics was performed as previously described[8]. Subsequently, shotgun metagenomic sequencing was performed (Clinical Microbiomics, Copenhagen, Denmark). Before sequencing, the quality of the DNA samples was evaluated using agarose gel electrophoresis, NanoDrop 2000 spectrophotometry and Qubit 2.0 fluorometer quantitation. The genomic DNA was randomly sheared into fragments of around 350 bp. The fragmented DNA was used for library construction using NEBNext Ultra Library Prep Kit for Illumina (New England Biolabs). The prepared DNA libraries were evaluated using Qubit 2.0 fluorometer quantitation and Agilent 2100 Bioanalyzer for the fragment size distribution. Real time quantitative PCR (qPCR) was used to determine the concentration of the final library before sequencing. The library was sequenced on an Illumina HiSeq platform to produce 2 x 150 bp paired-end reads. Raw reads were quality filtered using Trimmomatic (v0.38), removing adapters, trimming the first 5 bp, and then quality trimming reads using a sliding

window of 4 bp and a minimum Q-score of 15. Reads that were shorter than 70 bp after trimming were discarded. Surviving paired reads were mapped against the human genome (GRCh37_hg19) with bowtie2 (v2.3.4.3) in order to remove human reads. Finally, the remaining quality filtered, non-human reads were sub-sampled to 20 million reads per sample and processed using Metaphlan2[9] (v2.7.7) to infer metagenomic microbial species composition and Humann2[10] (v0.11.2) to extract gene counts and functional pathways. In brief, reads were mapped using bowtie2 against microbial pangenomes; unmapped reads were translated and mapped against the full Uniref90 protein database using diamond (v0.8.38). Pathway collection was performed using the MetaCyc database.

Small intestinal microbiota analyses

Biopsies were added to a bead-beating tube with 300 µl Stool Transport and Recovery (STAR) buffer, 0.25 g of sterilized zirconia beads (0.1 mm). 6 µl of Proteinase K (20mg/ml; QIAGEN, Venlo, The Netherlands) was added and incubated for 1hr at 55 °C. The biopsies were then homogenized by bead-beating three times (60 s × 5.5 ms) followed by incubation for 15 min at 95 °C at 1000 rpm. Samples were then centrifuged for 5 min at 4 °C and 14,000 g and supernatants transferred to sterile tubes. Pellets were re-processed using 200 µl STAR buffer and both supernatants were pooled. DNA purification was performed with a customized kit (AS1220; Promega) using 250 µl of the final supernatant pool. DNA was eluted in 50 µl of DNAse- RNAse-free water and its concentration measured using a DS-11 FX+ Spectrophotometer/Fluorometer (DeNovix Inc., Wilmington, USA) with the Qubit™ dsDNA BR Assay kit (Thermo Scientific, Landsmeer, The Netherlands). The V5-V6 region of 16S ribosomal RNA (rRNA) gene was amplified in duplicate PCR reactions for each sample in a total reaction volume of 50 µl. A first step PCR using the 27F and the 1369R primer were used for primary enrichment. 1µl of 10uM primer, 1 µl dNTPs mixture, 0.5 µl Phusion Green Hot Start II High-Fidelity DNA Polymerase (2 U/µl; Thermo Scientific, Landsmeer, The Netherlands), 10 µl 5× Phusion Green HF Buffer, and 36.5 µl DNAse- RNAse-free water. The amplification program included 30 s of initial denaturation step at 98°C, followed by 5 cycles of denaturation at 98 oC for 30 s, annealing at 52 °C

for 40 s, elongation at 72 °C for 90 s, and a final extension step at 72 °C for 7 min. On the PCR product a nested PCR was performed using the master mix containing 1 µl of a unique barcoded primer, 784F-n and 1064R-n (10 µM each per reaction), 1 µl dNTPs mixture, 0.5 µl Phusion Green Hot Start II High-Fidelity DNA Polymerase (2 U/µl; Thermo Scientific, Landsmeer, The Netherlands), 10 µl 5× Phusion Green HF Buffer, and 36.5 µl DNAse- RNAse-free water. The amplification program included 30 s of initial denaturation step at 98°C, followed by 5 cycles of denaturation at 98 °C for 10 s, annealing at 42 °C for 10 s, elongation at 72 °C for 10 s, and a final extension step at 72 °C for 7 min. The PCR product was visualised in 1% agarose gel (~280 bp) and purified with CleanPCR kit (CleanNA, Alphen aan den Rijn, The Netherlands). The concentration of the purified PCR product was measured with Qubit dsDNA BR Assay Kit (Invitrogen, California, USA) and 200 ng of microbial DNA from each sample were pooled for the creation of the final amplicon library which was sequenced (150 bp, paired-end) on the Illumina HiSeq. 2500 platform (GATC Biotech, Constance, Germany).

Raw reads were demultiplexed using the Je software suite (v2.0.) allowing no mismatches in the barcodes. After removing the barcodes, linker and primers, reads were mapped against the human genome using bowtie2 in order to remove human reads. Surviving microbial forward and reverse reads were pipelined separately using DADA2[11] (v1.12.1). Amplicon Sequence Variants (ASVs) inferred from the reverse reads were reverse-complemented and matched against ASVs inferred from the forwards reads. Only non-chimeric forward reads ASVs that matched reverse-complemented reverse reads ASVs were kept. ASV sample counts were inferred from the forward reads. ASV taxonomy was assigned using DADA2 and the SILVA (v132) database. The resulting ASV table and taxonomy assignments were integrated using the phyloseq R package (v1.28.0) and rarefied to 60000 counts per sample.

Duodenal gene expression

Fresh biopsy samples were snap frozen, stored at −80°C and processed as previously published (Pellegrini et al., 2017). Prior to RNA extraction, biopsies were transferred into 500 µl lysis buffer

(mirVana Isolation Kit, Ambion, Austin, TX), homogenized with Tissue Ruptor (Qiagen, Hilden, Germany) and frozen again. Total RNA was extracted with mirVana Kit following manufacturer's instruction and quantified by spectrophotometer lecture (Epoch, Gen5 software; BioTek, Winooski, VT). OD A260/A280 ratio ≥2.0 and *GAPDH* Ct<28 in Taqman single assay identified acceptable quality RNA samples. For reverse transcription PCR, after DNAse treatment (Turbo DNAse, Invitrogen), 5 µg of RNA were retro-transcribed in a 21 µl reaction volume with SuperScript IV RT (Invitrogen) following manufacturer's instructions. Predesigned TaqMan Arrays Human Inflammation Panel and Human Cell Junction Panel (Applied Biosystems, Foster City, CA) were used for gene expression study. A list of genes is reported in supplementary table 1. PCR runs and fluorescence detection were carried out in a 7900 Real-Time PCR System (Applied Biosystems) at the following temperature conditions: 50° C for 2 minutes, 95°C for 10 minutes and 40 cycles of 95° C for 15 seconds and 60° C for 1 minute. Results were expressed as fold changes (2^-ΔCt method) over a mean of expression of the selected best reference genes: 5 housekeeping (HK) genes for Human Inflammation panel l (β-*actin*, β-*2 Microglobulin*, *GAPDH, RPLP0* and *UBC)* and 4 housekeeping genes for Human Cell Junction Panel (β-*2 Microglobulin*, *GAPDH, RPLP0* and *UBC*).

Statistical analysis

For baseline differences between groups, unpaired Student's t-test or the Mann-Whitney U test (MWU) were used dependent on the distribution of the data. Accordingly, data are expressed as mean ± the standard deviation or the median with interquartile range. Post-prandial results (e.g. c-peptide) are described as area under the curves (AUC) for the 2-hour post-prandial follow-up, calculated by using the trapezoidal method. For correlation analyses, Spearman's Rank test was used (as all parameters were non-parametric). For comparison of the primary end point a linear mixed model (LMM) was used (lme4 package in R), where 'allocation' and 'time point' were fixed effects and 'patient entry number' was a random effect. The p value for the interaction between 'allocation' and 'time point' was reported. Additionally, parameters were compared between groups at various

time points using MWU with multiplicity correction. A p-value < 0.05 was considered statistically significant.

Missing values

One study participant retracted informed consent after the first visit. This participant was not included in our analyses. All other study participants completed all study visits, therefore missing values are limited. Most missing data points were caused by laboratory problems such as inability to extract DNA or failure to properly process or harvest immune cells. These missing data are considered to be missing completely at random (MCAR). The exception to this is that one subject refused the second gastroduodenoscopy, therefore his duodenal biopsies (small intestinal microbiota and gene expression) after treatment are missing (1 in 20 cases or 5%). This subject has received autologous FMT. We do not assume that having received autologous treatment rather than allogenic (donor) faeces, metabolism or gene expression are in any way related to this person refusing the second gastroscopy, therefore we consider these data to be 'missing at random'(MAR). Key variables fasting C-peptide, C-peptide AUC, A1c and weight are complete (0% missing). The immunological parameters mentioned in the text and figures (main figure 6 and supplementary figure 3) are all based on complete data sets i.e. no missing values (CD4+ CM T cells, CD8+ T cells, CD8+CXCR3+ T cells and CD4+CXCR3+ T cells). Most gene expression data in the manuscript and main and supplemental figures (CCL22, CLDN12, CCL4, CD86, CCL19, CLDN 14, CCR5, CCL18, CD14) is 95% complete (see above). For CCL13 one extra baseline measurement is missing, for CXCL12 one 'after treatment' time point is missing, for CXCL1 two baseline and 1 after treatment time point is missing. Some immunological analyses have suffered from missing data, e.g. the lymphocyte stimulation tests (LST) analyses (1 to 4/20 (5-20%) of cases depending on the parameter). However, these data are not mentioned in the figures (there was no statistically significant difference between the groups). The fecal microbiota dataset is complete (complete case analysis). The missing values in the metabolite

data were imputated (see paragraph on metabolite analysis), therefore complete case analysis was performed. No other data have been imputated.

Machine learning and follow-up statistical analyses

This technique was used on duodenal microbial composition (perform RT-qPCR on biopsies), on fecal microbiota composition and metabolic pathway abundance (Shotgun sequencing), on plasma metabolite levels and on duodenal gene expression levels data. To predict treatment groups, we used the relative change (delta) of each parameter between 0 and 12 months. For duodenal microbes and duodenal gene expression, we used delta 0 vs 6 months as no 12 months' time point was available. For prediction of responders vs non-responders baseline values, delta 0 vs 6 months and delta 0 vs 12 months were used. Each analysis produced a ranked list of the top 30 most discriminative features. We selected the top parameters from each analysis that accurately (i.e., areau under the receiver-operator curve (AUROC)≥0.8) or moderately (AUROC > 0.7) predicted group allocation for closer study, using an arbitrary cut off. This cut off was generally a relative importance of around 30% or higher (for an example of this see figure 2C, from which the top 4 features were selected). Then, we visualized the change in time of the selected parameters (Wilcoxon's signed rank tests) and studied between-group differences (MWU) at each time point and finally, using Spearman's rank test, we correlated these parameters with our primary end point and with other key parameters that were identified in this way. For the most important analyses supplementary figures showing the top 30 selected features are presented.

Analysis of responders and non-responders irrespective of treatment group

We investigated whether baseline characteristics of T1D patients can predict response to FMT therapy at 12 months follow-up and which bacterial strains and plasma metabolites were associated with this response. Clinical response was defined as <10% decline in Beta-cell function compared to baseline at 12 months follow-up, which is significantly less than the expected natural 12 months decline of 20% in beta cell function [4,12]. We chose responders at 12 months for our analyses because our primary end point (MMT stimulated C-peptide) was significantly different at 12 (but not at 6) months. At 12 months follow-up, clinical response sustained in 10 subjects of whom 3 had received allogenic and 7 had received autologous FMT (see Figure 4A-B). We next used predictive modelling to determine which parameters (either their baseline values or delta 0-12 month values) were predictors of clinical response to FMT.

Patient and public involvement

This research was done without patient involvement. Patients were not invited to comment on the study design and were not consulted to develop patient relevant outcomes or interpret the results. Patients were not invited to contribute to the writing or editing of this document for readability or accuracy.

References

1 van Nood E, Vrieze A, Nieuwdorp M, *et al.* Duodenal infusion of donor feces for recurrent Clostridium difficile. *N Engl J Med* 2013;**368**:407–15. doi:10.1056/NEJMoa1205037

2 Cammarota G, Ianiro G, Tilg H, *et al.* European consensus conference on faecal microbiota transplantation in clinical practice. *Gut* 2017;**66**:569–80. doi:10.1136/gutjnl-2016-313017

3 Moran A, Bundy B, Becker DJ, *et al.* Interleukin-1 antagonism in type 1 diabetes of recent onset: two multicentre, randomised, double-blind, placebo-controlled trials. *Lancet (London,*

England) 2013;**381**:1905–15. doi:10.1016/S0140-6736(13)60023-9

4 Lachin JM, McGee PL, Greenbaum CJ, *et al.* Sample size requirements for studies of treatment effects on beta-cell function in newly diagnosed type 1 diabetes. *PLoS One* 2011;**6**:e26471. doi:10.1371/journal.pone.0026471

5 Kracht MJL, van Lummel M, Nikolic T, *et al.* Autoimmunity against a defective ribosomal insulin gene product in type 1 diabetes. *Nat Med* 2017;**23**:501–7. doi:10.1038/nm.4289

6 Velthuis JH, Unger WW, Abreu JRF, *et al.* Simultaneous detection of circulating autoreactive CD8+ T-cells specific for different islet cell-associated epitopes using combinatorial MHC multimers. *Diabetes* 2010;**59**:1721–30. doi:10.2337/db09-1486

7 Koh A, Molinaro A, Stahlman M, *et al.* Microbially Produced Imidazole Propionate Impairs Insulin Signaling through mTORC1. *Cell* 2018;**175**:947-961.e17. doi:10.1016/j.cell.2018.09.055

8 Vrieze A, Van Nood E, Holleman F, *et al.* Transfer of intestinal microbiota from lean donors increases insulin sensitivity in individuals with metabolic syndrome. *Gastroenterology* 2012;**143**:913-6.e7. doi:10.1053/j.gastro.2012.06.031

9 Truong DT, Franzosa EA, Tickle TL, *et al.* MetaPhlAn2 for enhanced metagenomic taxonomic profiling. Nat. Methods. 2015;**12**:902–3. doi:10.1038/nmeth.3589

10 Franzosa EA, McIver LJ, Rahnavard G, *et al.* Species-level functional profiling of metagenomes and metatranscriptomes. *Nat Methods* 2018;**15**:962–8. doi:10.1038/s41592-018-0176-y

11 Callahan BJ, McMurdie PJ, Rosen MJ, *et al.* DADA2: High-resolution sample inference from Illumina amplicon data. *Nat Methods* 2016;**13**:581–3. doi:10.1038/nmeth.3869

12 Overgaard AJ, Weir JM, Jayawardana K, *et al.* Plasma lipid species at type 1 diabetes onset predict residual beta-cell function after 6 months. *Metabolomics* 2018;**14**:158. doi:10.1007/s11306-018-1456-3

BMJ Publishing Group Limited (BMJ) disclaims all liability and responsibility arising from any reliance placed on this supplemental material which has been supplied by the author(s)
