# Supplementary material for: Faecal microbiota transplantation halts progression of human new-onset type 1 diabetes in a randomised controlled trial
Source: Gut. 2020 Oct 26;70(1):92–105. doi: 10.1136/gutjnl-2020-322630 (PMC7788262; doi:10.1136/gutjnl-2020-322630)
Supplement: Supplementary data [file gutjnl-2020-322630supp002.pdf]

Supplementary table 1

Genecard Cell Junctions			Genecard Inflammation		
Gene code	Gene name	Gene type	Gene code	Gene name	Gene type
GAPDH-Hs99999905_m1	glyceraldehyde-3-phosphate dehydrogenase	housekeeping	GAPDH-Hs99999905_m1	glyceraldehyde-3-phosphate dehydrogenase	housekeeping
CAV1-Hs00971716_m1	caveolin 1	target	ACTB-Hs99999903_m1	actin beta	housekeeping
CAV2-Hs00184597_m1	caveolin 2	target	ALOX5-Hs00167536_m1	arachidonate 5-lipoxygenase	target
CAV3-Hs00154292_m1	caveolin 3	target	B2M-Hs99999907_m1	beta-2-microglobulin	housekeeping
CDH1-Hs01023894_m1	cadherin 1	target	HSPA5-Hs00607129_gH	heat shock protein family A (Hsp70) member 5	target
CDH2-Hs00983056_m1	cadherin 2	target	CARD9-Hs00364485_m1	caspase recruitment domain family member 9	target
CLDN1-Hs00221623_m1	claudin 1	target	ACKR2-Hs00174299_m1	atypical chemokine receptor 2	target
CLDN10-Hs00734479_m1	claudin 10	target	CCL11-Hs00237013_m1	C-C motif chemokine ligand 11	target
CLDN11-Hs00194440_m1	claudin 11	target	CCL13-Hs00234646_m1	C-C motif chemokine ligand 13	target
CLDN12-Hs00273258_s1	claudin 12	target	CCL15-CCL14;CCL15-Hs00263142_m1	CCL15-CCL14, C-C motif chemokine ligand 15	target
CLDN14-Hs00273267_s1	claudin 14	target	CCL16-Hs00171123_m1	C-C motif chemokine ligand 16	target
CLDN15-Hs00204982_m1	claudin 15	target	CCL17-Hs00171074_m1	C-C motif chemokine ligand 17	target
CLDN16-Hs01070692_m1	claudin 16	target	CCL18-Hs00268113_m1	C-C motif chemokine ligand 18	target
CLDN17-Hs01043467_s1	claudin 17	target	CCL19-Hs00171149_m1	C-C motif chemokine ligand 19	target
CLDN18-Hs00212584_m1	claudin 18	target	CCL1-Hs00171072_m1	C-C motif chemokine ligand 1	target
CLDN19-Hs00961709_m1	claudin 19	target	CCL2-Hs00234140_m1	C-C motif chemokine ligand 2	target
CLDN2-Hs00252666_s1	claudin 2	target	CCL20-Hs00171125_m1	C-C motif chemokine ligand 20	target
CLDN3-Hs00265816_s1	claudin 3	target	CCL21-Hs00171076_m1	C-C motif chemokine ligand 21	target
CLDN4-Hs00976831_s1	claudin 4	target	CCL22-Hs00171080_m1	C-C motif chemokine ligand 22	target
CLDN5-Hs00533949_s1	claudin 5	target	CCL25-Hs00171144_m1	C-C motif chemokine ligand 25	target
CLDN6-Hs00607528_s1	claudin 6	target	CCL26-Hs00171146_m1	C-C motif chemokine ligand 26	target
CLDN7-Hs00600772_m1	claudin 7	target	UBC-Hs00824723_m1	ubiquitin C	housekeeping
CLDN8-Hs04186769_s1	claudin 8	target	CCL3-Hs00234142_m1	C-C motif chemokine ligand 3	target
CLDN9-Hs00253134_s1	claudin 9	target	CCL4-Hs99999148_m1	C-C motif chemokine ligand 4	target
DLL1-Hs00194509_m1	delta like canonical Notch ligand 1	target	CCL5-Hs00174575_m1	C-C motif chemokine ligand 5	target
DSC1-Hs00245189_m1	desmocollin 1	target	CCL7-Hs00171147_m1	C-C motif chemokine ligand 7	target
DSC2-Hs00951428_m1	desmocollin 2	target	CCL8-Hs00271615_m1	C-C motif chemokine ligand 8	target
DSC3-Hs00170032_m1	desmocollin 3	target	CCR1-Hs00174298_m1	C-C motif chemokine receptor 1	target
DSG1-Hs00355084_m1	desmoglein 1	target	CCR2-Hs00356601_m1	C-C motif chemokine receptor 2	target
DSG2-Hs00170071_m1	desmoglein 2	target	CCR3-Hs00266213_s1	C-C motif chemokine receptor 3	target
DSG3-Hs00951897_m1	desmoglein 3	target	CCR4-Hs99999919_m1	C-C motif chemokine receptor 4	target
DSG4-Hs00698286_m1	desmoglein 4	target	CCR5-Hs00152917_m1	C-C motif chemokine receptor 5	target
DSP-Hs00950591_m1	desmoplakin	target	CCR6-Hs00171121_m1	C-C motif chemokine receptor 6	target
DST-Hs00156137_m1	dystonin	target	CCR7-Hs00171054_m1	C-C motif chemokine receptor 7	target
ESAM-Hs00332781_m1	endothelial cell adhesion molecule	target	CCR8-Hs00174764_m1	C-C motif chemokine receptor 8	target
F11R-Hs00170991_m1	F11 receptor	target	CD14-Hs02621496_s1	CD14 molecule	target
GJA1-Hs00748445_s1	gap junction protein alpha 1	target	CD28-Hs01007422_m1	CD28 molecule	target
GJA3-Hs00254296_s1	gap junction protein alpha 3	target	CD68-Hs00154355_m1	CD68 molecule	target
GJA4-Hs00704917_s1	gap junction protein alpha 4	target	CD80-Hs01045161_m1	CD80 molecule	target
GJA5-Hs00270952_s1	gap junction protein alpha 5	target	CD86-Hs01567026_m1	CD86 molecule	target
GJA8-Hs00270960_s1	gap junction protein alpha 8	target	CHGA-Hs00900375_m1	chromogranin A	target
GJB1-Hs00939759_s1	gap junction protein beta 1	target	DDIT3-Hs00358796_g1	DNA damage inducible transcript 3	target
GJB2-Hs00269615_s1	gap junction protein beta 2	target	PTGS2-Hs00153133_m1	prostaglandin-endoperoxide synthase 2	target
GJB3-Hs02378125_s1	gap junction protein beta 3	target	CSF1-Hs00174164_m1	colony stimulating factor 1	target
GJB4-Hs00920816_s1	gap junction protein beta 4	target	CTLA4-Hs00175480_m1	cytotoxic T-lymphocyte associated protein 4	target
GJB5-Hs01921450_s1	gap junction protein beta 5	target	CX3CL1-Hs00171086_m1	C-X3-C motif chemokine ligand 1	target
GJB6-Hs00922742_s1	gap junction protein beta 6	target	CX3CR1-Hs00365842_m1	C-X3-C motif chemokine receptor 1	target
GJC2-Hs00252713_s1	gap junction protein gamma 2	target	CXCL10-Hs00171042_m1	C-X-C motif chemokine ligand 10	target
GJD2-Hs00950432_m1	gap junction protein delta 2	target	CXCL12-Hs00171022_m1	C-X-C motif chemokine ligand 12	target
GJC3-Hs01384570_m1	gap junction protein gamma 3	target	CXCL1-Hs00236937_m1	C-X-C motif chemokine ligand 1	target
ICAM1-Hs00164932_m1	intercellular adhesion molecule 1	target	CXCL9-Hs00171065_m1	C-X-C motif chemokine ligand 9	target
ICAM2-Hs00609563_m1	intercellular adhesion molecule 2	target	CXCR1-Hs00174146_m1	C-X-C motif chemokine receptor 1	target
ITGA1-Hs00235006_m1	integrin subunit alpha 1	target	CXCR2-Hs00174304_m1	C-X-C motif chemokine receptor 2	target
ITGA2-Hs00158127_m1	integrin subunit alpha 2	target	CXCR3-Hs00171041_m1	C-X-C motif chemokine receptor 3	target
ITGA3-Hs01076873_m1	integrin subunit alpha 3	target	CXCR4-Hs00237052_m1	C-X-C motif chemokine receptor 4	target
ITGA4-Hs00168433_m1	integrin subunit alpha 4	target	CXCR6-Hs00174843_m1	C-X-C motif chemokine receptor 6	target
ITGA5-Hs01547673_m1	integrin subunit alpha 5	target	ACKR3-Hs00604567_m1	atypical chemokine receptor 3	target
ITGA6-Hs01041011_m1	integrin subunit alpha 6	target	FCGR3B;FCGR3A-Hs00275547_m1	Fc fragment of IgG receptor IIIb,Fc fragment of IgG receptor IIIa	target
ITGA7-Hs00174397_m1	integrin subunit alpha 7	target	GAD2-Hs00609534_m1	glutamate decarboxylase 2	target
ITGA8-Hs00233321_m1	integrin subunit alpha 8	target	HCK-Hs01067403_m1	HCK proto-oncogene, Src family tyrosine kinase	target
ITGA9-Hs00979865_m1	integrin subunit alpha 9	target	PTPRN-Hs01090891_g1	protein tyrosine phosphatase, receptor type N	target
ITGAL-Hs00158218_m1	integrin subunit alpha L	target	IDO1-Hs00984148_m1	indoleamine 2,3-dioxygenase 1	target
ITGAM-Hs00355885_m1	integrin subunit alpha M	target	IFNG-Hs00174143_m1	interferon gamma	target
ITGAV-Hs00233808_m1	integrin subunit alpha V	target	IL10-Hs00174086_m1	interleukin 10	target
ITGB1-Hs00559595_m1	integrin subunit beta 1	target	IL12A-Hs00168405_m1	interleukin 12A	target
ITGB2-Hs00164957_m1	integrin subunit beta 2	target	RPLP0-Hs99999902_m1	ribosomal protein lateral stalk subunit P0	housekeeping
ITGB3-Hs01001469_m1	integrin subunit beta 3	target	IL12B-Hs00233688_m1	interleukin 12B	target
ITGB4-Hs00236216_m1	integrin subunit beta 4	target	IL15-Hs00542562_m1	interleukin 15	target
ITGB5-Hs00174435_m1	integrin subunit beta 5	target	IL15RA-Hs00542602_g1	interleukin 15 receptor subunit alpha	target
ITGB6-Hs00168458_m1	integrin subunit beta 6	target	IL17A-Hs00174383_m1	interleukin 17A	target
JAM2-Hs01022006_m1	junctional adhesion molecule 2	target	IL1R1-Hs00991010_m1	interleukin 1 receptor type 1	target
JAM3-Hs00230289_m1	junctional adhesion molecule 3	target	IL1B-Hs00174097_m1	interleukin 1 beta	target
JUP-Hs00158408_m1	junction plakoglobin	target	IL2-Hs00174114_m1	interleukin 2	target
NOTCH1-Hs01062014_m1	notch 1	target	IL22-Hs01574154_m1	interleukin 22	target
NOTCH2-Hs01050702_m1	notch 2	target	IL4-Hs00174122_m1	interleukin 4	target
NOTCH3-Hs01128541_m1	notch 3	target	IL4R-Hs00166237_m1	interleukin 4 receptor	target
NOTCH4-Hs00965889_m1	notch 4	target	IL6-Hs00174131_m1	interleukin 6	target
OCLN-Hs00170162_m1	occludin	target	CXCL8-Hs00174103_m1	C-X-C motif chemokine ligand 8	target
PLEC-Hs00356986_g1	plectin	target	INS-Hs02741908_m1	insulin	target
NECTIN1-Hs01591978_m1	nectin cell adhesion molecule 1	target	LAG3-Hs00958444_g1	lymphocyte activating 3	target
NECTIN2-Hs01071562_m1	nectin cell adhesion molecule 2	target	LST1-Hs00705788_s1	leukocyte specific transcript 1	target
NECTIN3-Hs00210043_m1	nectin cell adhesion molecule 3	target	CIITA-Hs00172106_m1	class II, major histocompatibility complex, transactivator	target
TJP1-Hs01551861_m1	tight junction protein 1	target	MIF-Hs00236988_g1	macrophage migration inhibitory factor	target
TJP2-Hs00910543_m1	tight junction protein 2	target	NOD2-Hs00223394_m1	nucleotide binding oligomerization domain containing 2	target
TJP3-Hs00274276_m1	tight junction protein 3	target	NOS2-Hs00167257_m1	nitric oxide synthase 2	target
HPRT1-Hs99999909_m1	hypoxanthine phosphoribosyltransferase 1	housekeeping	PDCD1-Hs01550088_m1	programmed cell death 1	target
GUSB-Hs99999908_m1	glucuronidase beta	housekeeping	CD274-Hs00204257_m1	CD274 molecule	target
ACTB-Hs99999903_m1	actin beta	housekeeping	PTX3-Hs00173615_m1	pentraxin 3	target
B2M-Hs99999907_m1	beta-2-microglobulin	housekeeping	SIGIRR-Hs00222347_m1	single Ig and TIR domain containing	target
HMBS-Hs00609297_m1	hydroxymethylbilane synthase	housekeeping	TMEM173-Hs00736955_g1	transmembrane protein 173	target
IPO8-Hs00183533_m1	importin 8	housekeeping	SYP-Hs00300531_m1	synaptophysin	target
PGK1-Hs99999906_m1	phosphoglycerate kinase 1	housekeeping	TNF-Hs00174128_m1	tumor necrosis factor	target
RPLP0-Hs99999902_m1	ribosomal protein lateral stalk subunit P0	housekeeping	TSPAN7-Hs00190284_m1	tetraspanin 7	target
TBP-Hs99999910_m1	TATA-box binding protein	housekeeping	VEGFA-Hs00900054_m1	vascular endothelial growth factor A	target
TFRC-Hs99999911_m1	transferrin receptor	housekeeping	C10orf54-Hs00735289_m1	chromosome 10 open reading frame 54	target
UBC-Hs00824723_m1	ubiquitin C	housekeeping	KCNJ8-Hs00958961_m1	potassium voltage-gated channel subfamily J member 8	target
