# Supplementary material for: Faecal microbiota transplantation halts progression of human new-onset type 1 diabetes in a randomised controlled trial
Source: Gut. 2020 Oct 26;70(1):92–105. doi: 10.1136/gutjnl-2020-322630 (PMC7788262; doi:10.1136/gutjnl-2020-322630)
Supplement: Supplementary data [file gutjnl-2020-322630supp003.pdf]

BMJ Publishing Group Limited (BMJ) disclaims all liability and responsibility arising from any reliance placed on this supplemental material which has been supplied by the author(s)

Parameter	Groups	Delta or baseline	AUC ± CI	1st most predictive variable	2nd	3rd
Metabolites	Tx groups	Δ 0 – 12M	0.79 ± 0.23	1-myristoyl-2-arachidonoyl-GPC	1-(1-enyl-palmitoyl)-2-linoleoyl-GPE	1-arachidonoyl-GPC
	R12	Baseline	0.70 ± 0.28	7-hydroxyoctanoate	N-acetylphenylalanine	2-methylcitrate/homocitrate
		Δ 0 – 12M	0.74 ± 0.25	7-hydroxyoctanoate	14 or 15-methylpalmitate	5-methylthioadenosine
Small intestinal microbes	Tx groups	Δ 0 – 12M	0.89 ± 0.18	Prevotella 1	Prevotella 2	Streptococcus oralis
	R12	Baseline	0.72 ± 0.27	Undibacterium oligocarboniphilum	Nesterenkonia flava	Shewanella colwelliana
		Δ 0 – 6M	0.60 ± 0.29	Neisseria animalis	Tenuibacillus multivorans	Streptococcus mitis
Fecal microbes (taxonomy)	Tx groups	Δ 0 – 6M	0.58 ± 0.24	Desulfovibrio piger	Bacteroidales bacterium ph8	Ruminococcus callidus
		Δ 0 – 12M	0.72 ± 0.24	Desulfovibrio piger	Eubacterium ventriosum	Sutterella wadsworthensis
	R12	Baseline	0.93 ± 0.14	Coprococcus catus	Bacteroides caccae	Paraprevotella unclassified
		Δ 0 – 6M	0.78 ± 0.23	Lachnospiraceae bacterium 8 1 57FAA	Collinsella aerofaciens	Holdemania unclassified
		Δ 0 – 12M	0.76 ± 0.23	Bacteroidales bacterium ph8	Actinomyces viscosus	Bacteroides thetaiotaomicron
Fecal microbes (metabolic pathways)	Tx groups	Δ 0 – 6M	0.75 ± 0.24	GDP-mannose biosynthesis	dTDP-L-rhamnose biosynthesis I	seleno-amino acid biosynthesis
		Δ 0 – 12M	0.68 ± 0.27	seleno-amino acid biosynthesis	UMP biosynthesis	superpathway of UDP-glucose-derived O-antigen building blocks biosynthesis
	R12	Baseline	0.85 ± 0.22	fatty acid β-oxidation I	pyruvate fermentation to acetone	colanic acid building blocks biosynthesis
		Δ 0 – 6M	0.70 ± 0.27	glycogen biosynthesis I (from ADP-D-Glucose)	phosphatidylcholine acyl editing	L-lysine biosynthesis II
		Δ 0 – 12M	0.69 ± 0.22	creatinine degradation I	Bifidobacterium shunt	glycolysis III (from glucose)
Duodenal gene expression	Tx groups	Δ 0 – 6M	0.61 ± 0.24	CCL18	CXCR1	CXCR4
	R12	Baseline	0.83 ± 0.21	CCL22	CLDN12	CCL4
		Δ 0 – 6M	0.73 ± 0.24	CCR5	CCL18	CD14

de Groot P, *et al. Gut* 2020; 70:92–105. doi: 10.1136/gutjnl-2020-322630

Supplementary table 2: AUCs. This table provides an overview of all predictive modeling analyses that we have performed. It shows what parameter was studied, in which group the analysis was done, whether baseline or delta values were used, how well the predictive model performed (measured asAUROC) and what were the top 3 predictive parameters from that analysis. The highest AUC from each category in bold. Tx: treatment, R12: responders versus non-responders at 12 months, Baseline: for this analysis, the baseline value of the parameters were used, Δ 0 – 12M: for this analysis, the delta's between baseline and 12 months were used. AUROC: area under the receiver-operator curve ± confidence interval.
