# Supplementary figures and images for: Faecal microbiota transplantation halts progression of human new-onset type 1 diabetes in a randomised controlled trial
Source: Gut. 2020 Oct 26;70(1):92–105. doi: 10.1136/gutjnl-2020-322630 (PMC7788262; doi:10.1136/gutjnl-2020-322630)

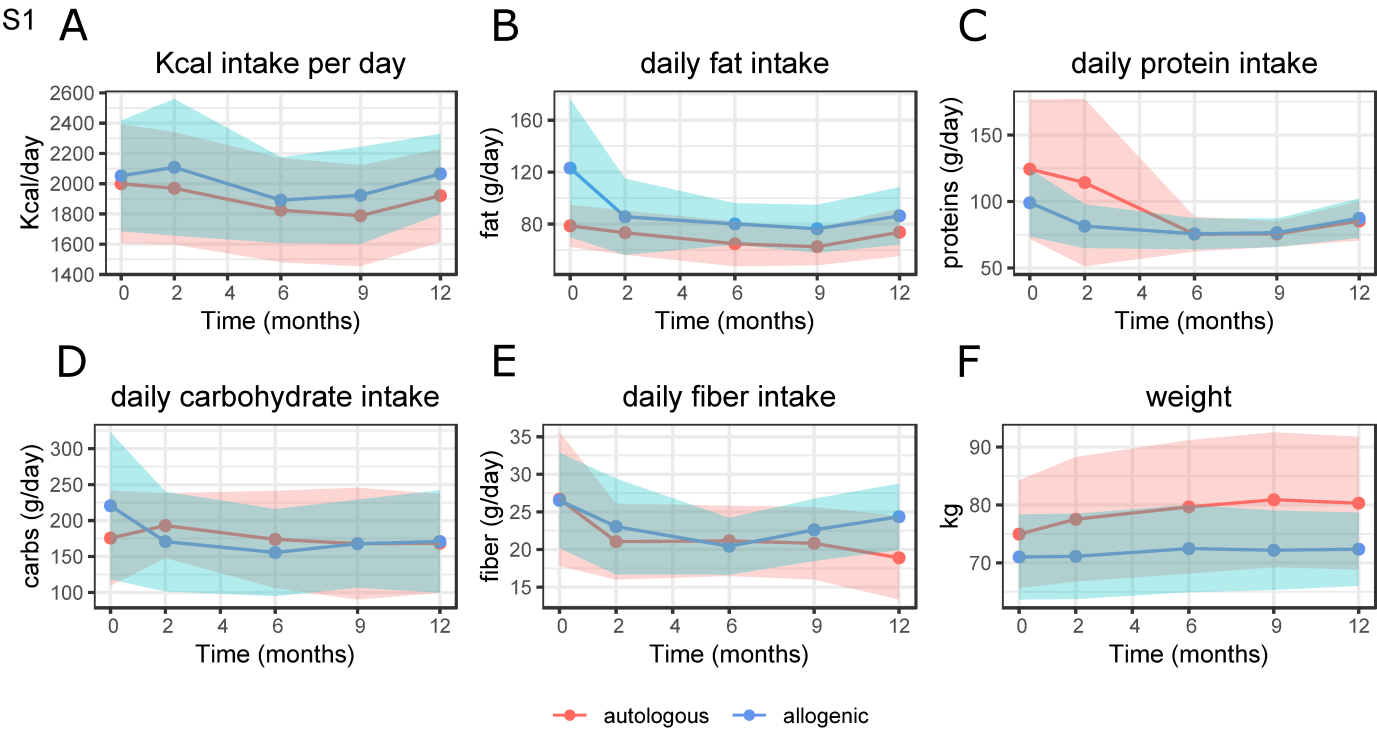

S2

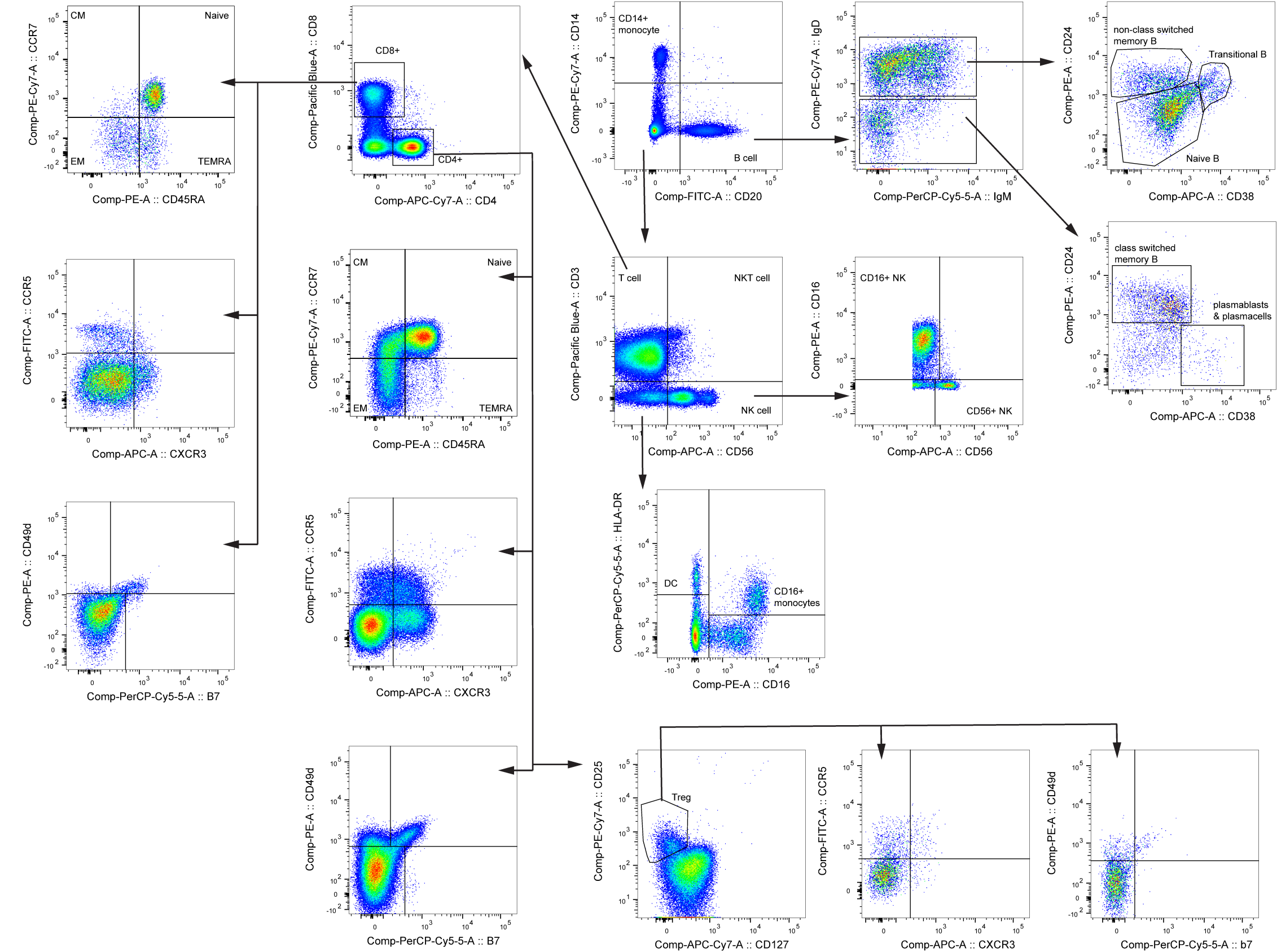

S3 A

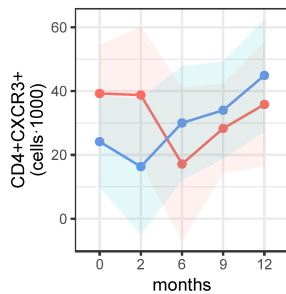

B

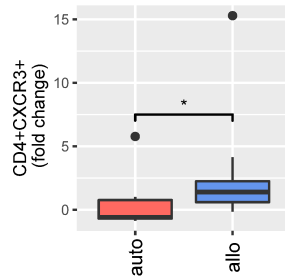

C

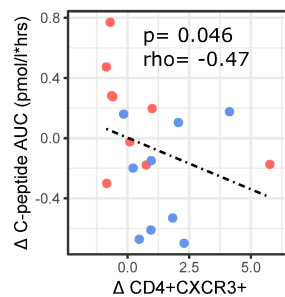

D

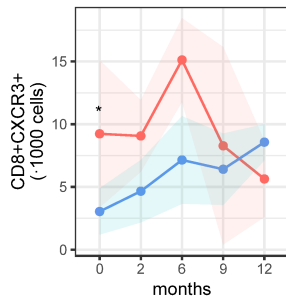

E

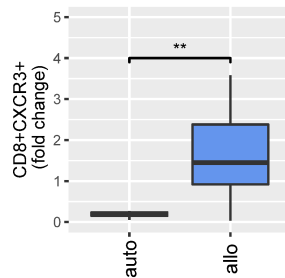

F

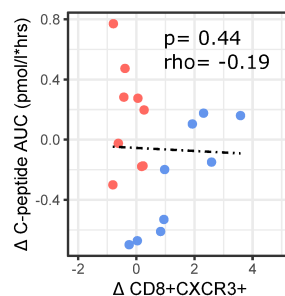

S4

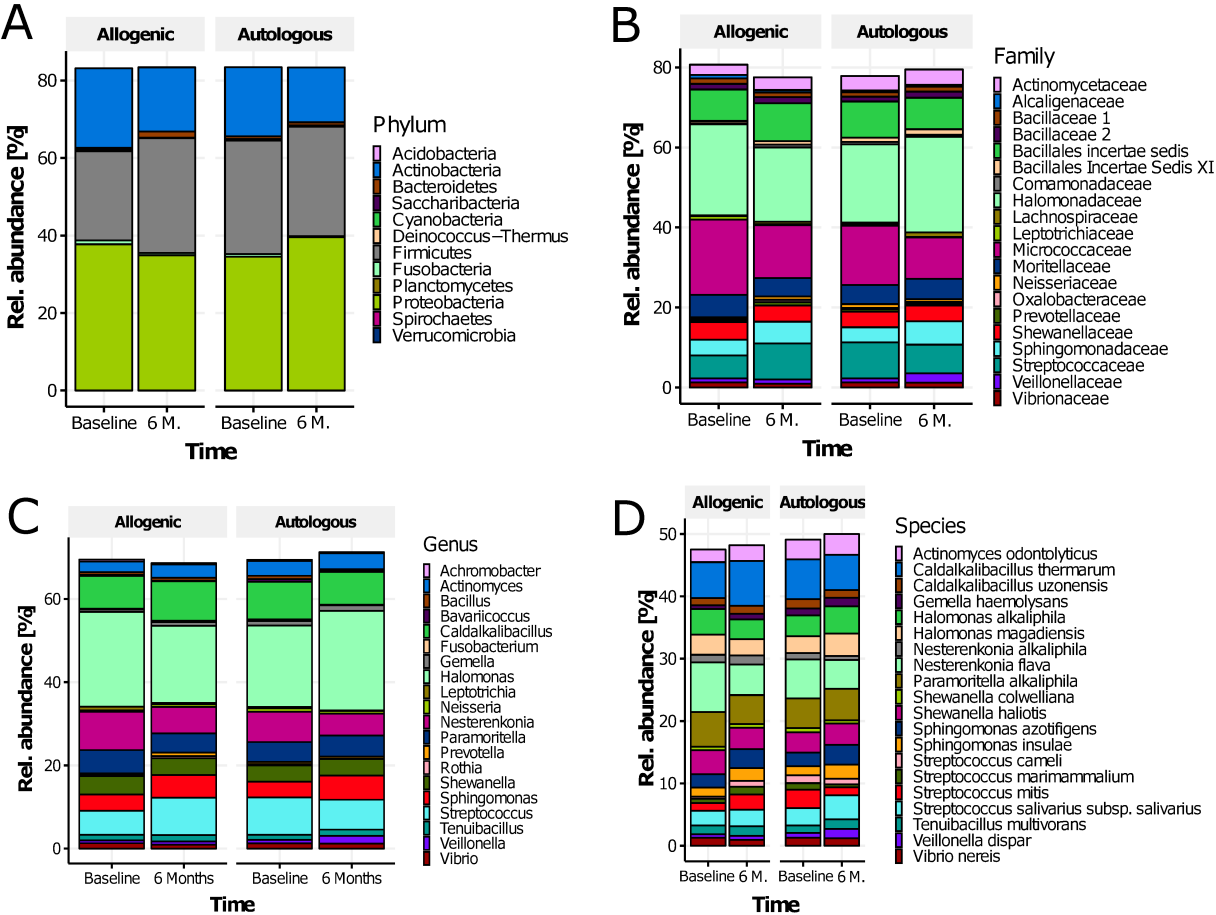

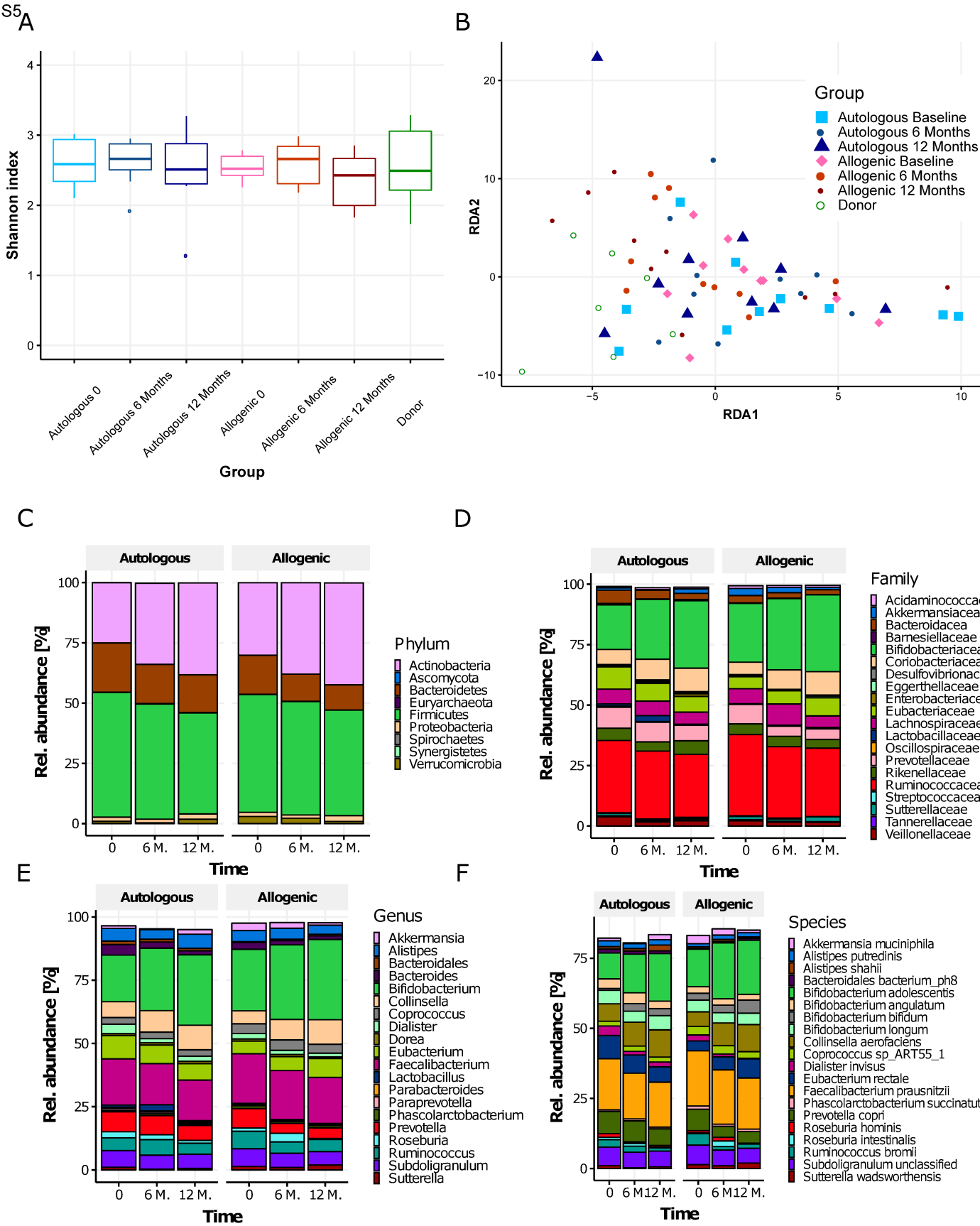

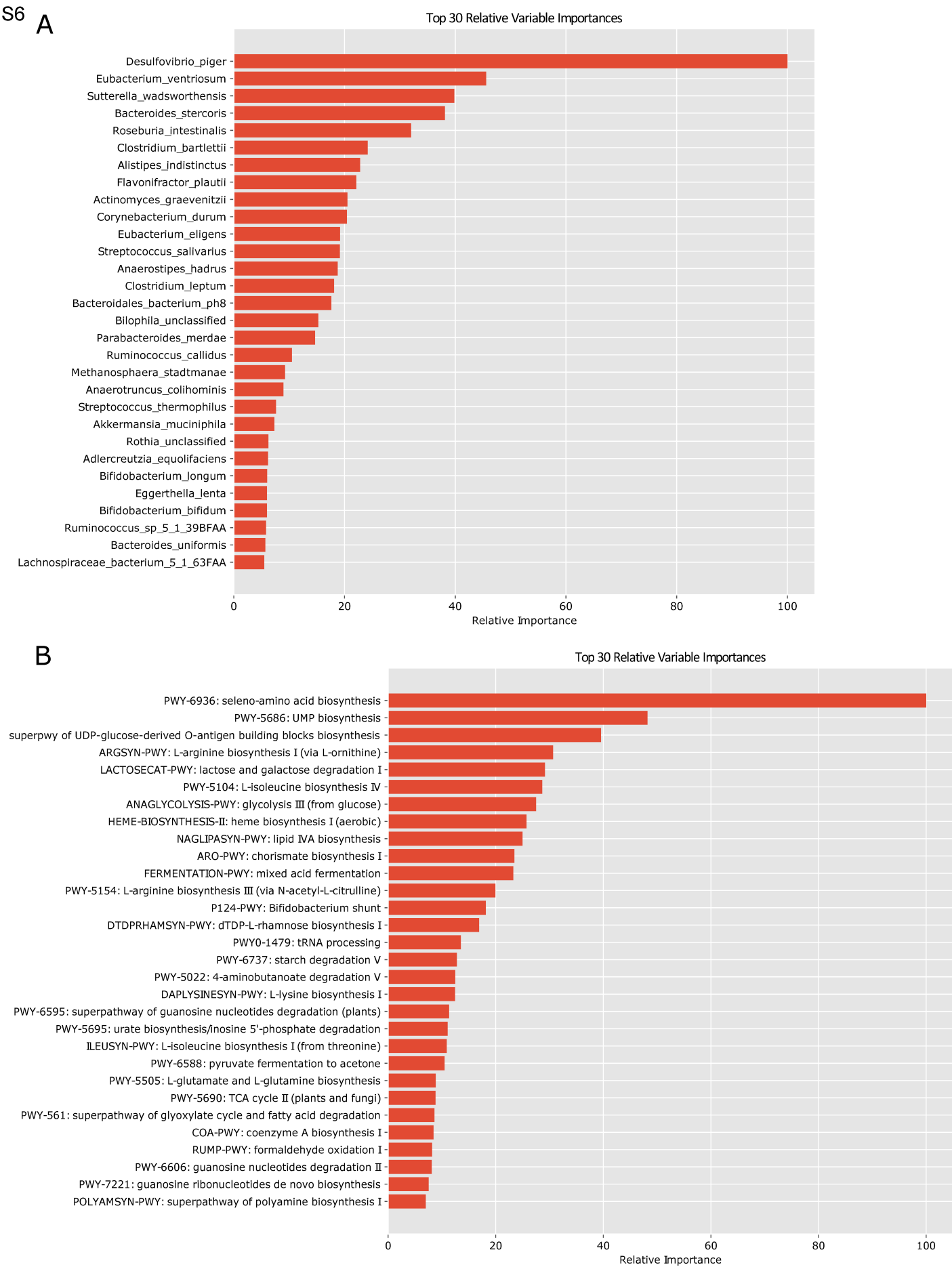

S7

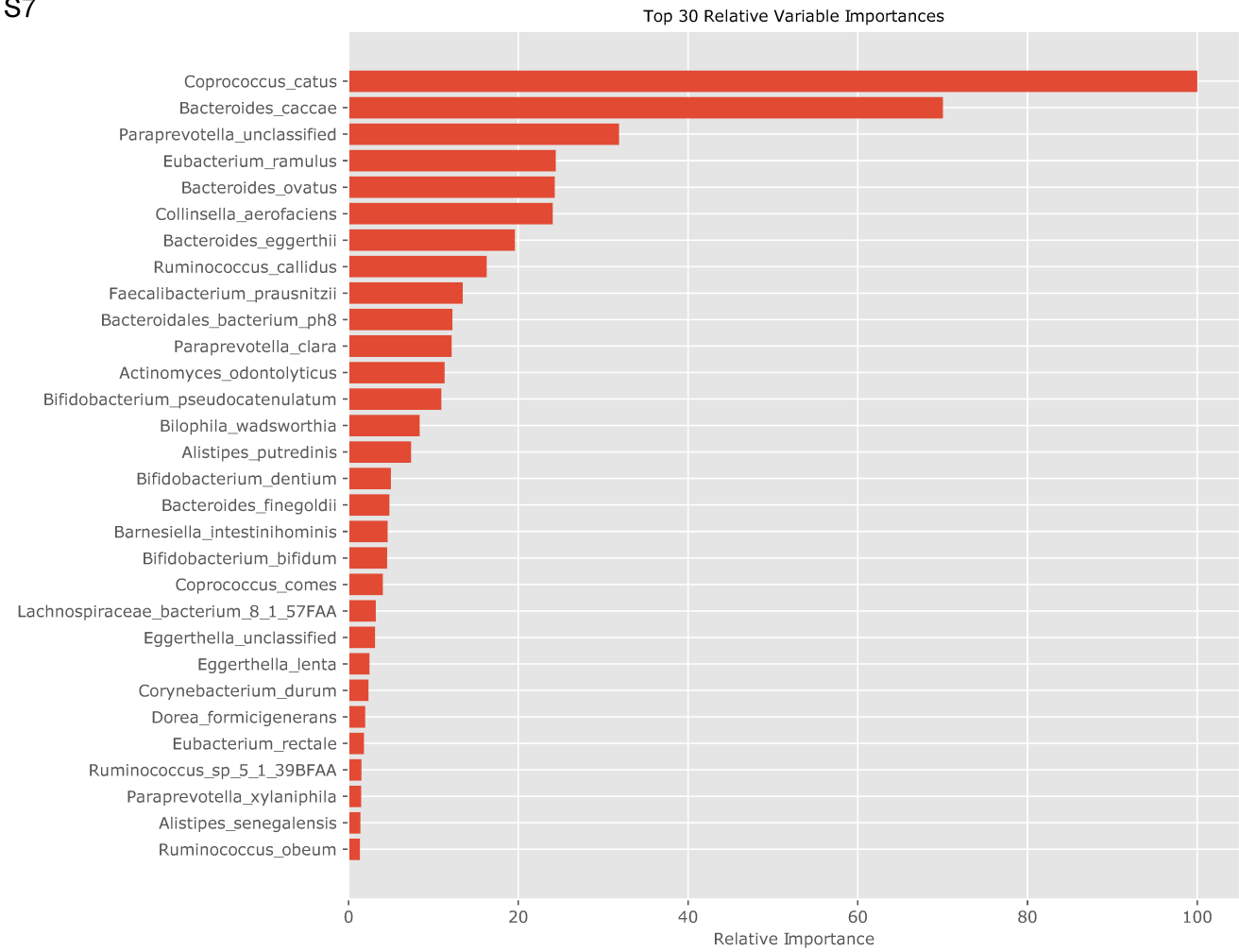

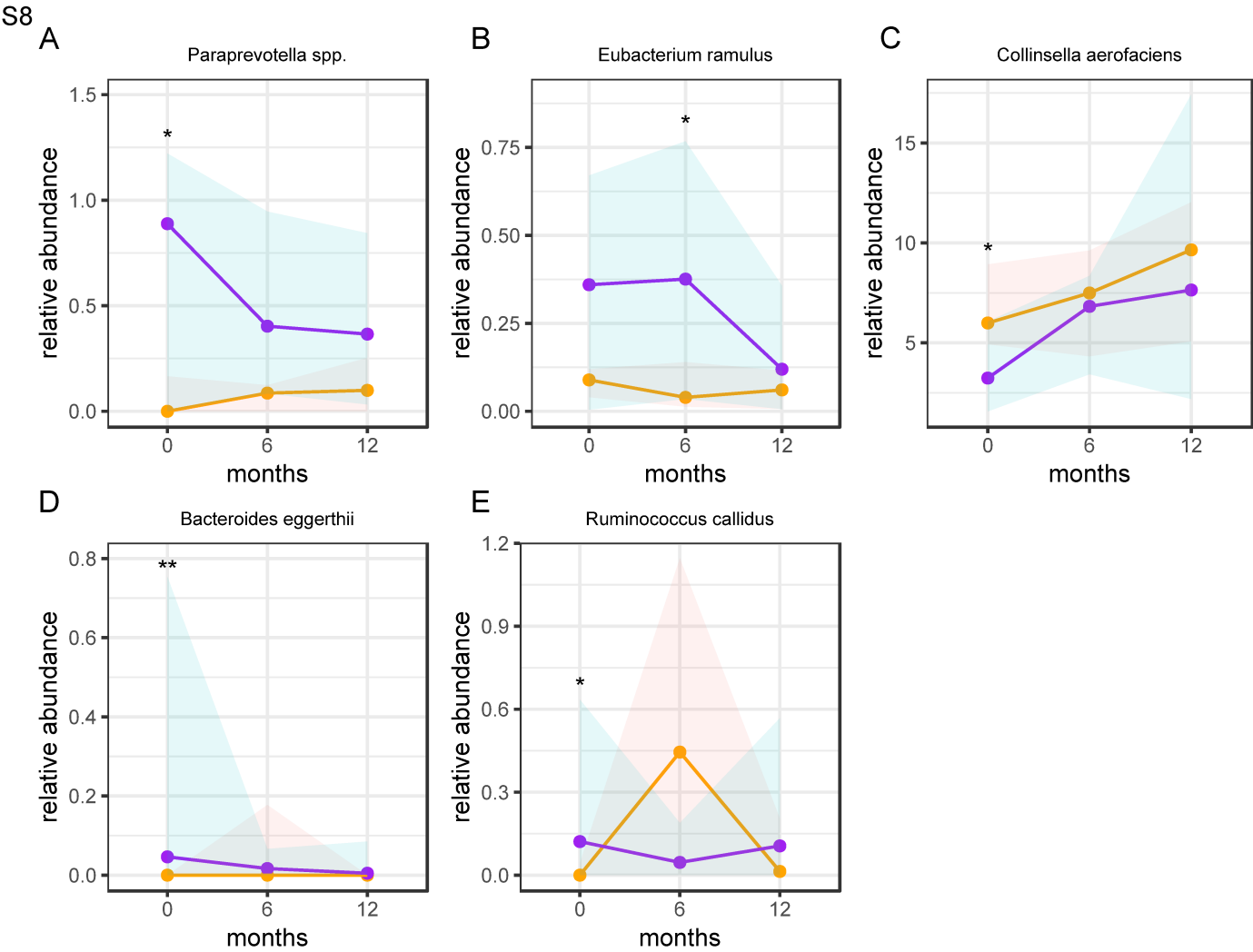

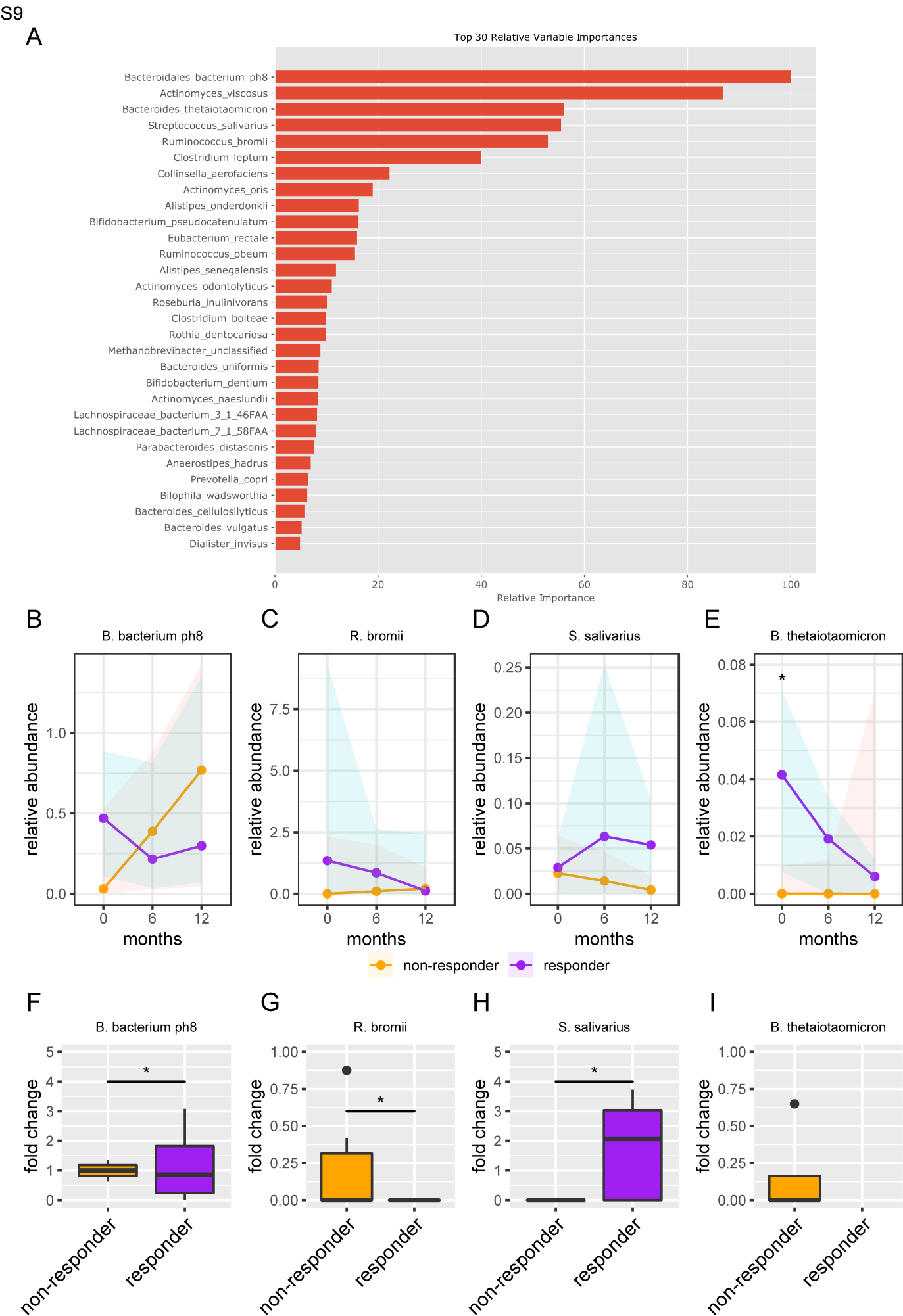

S10

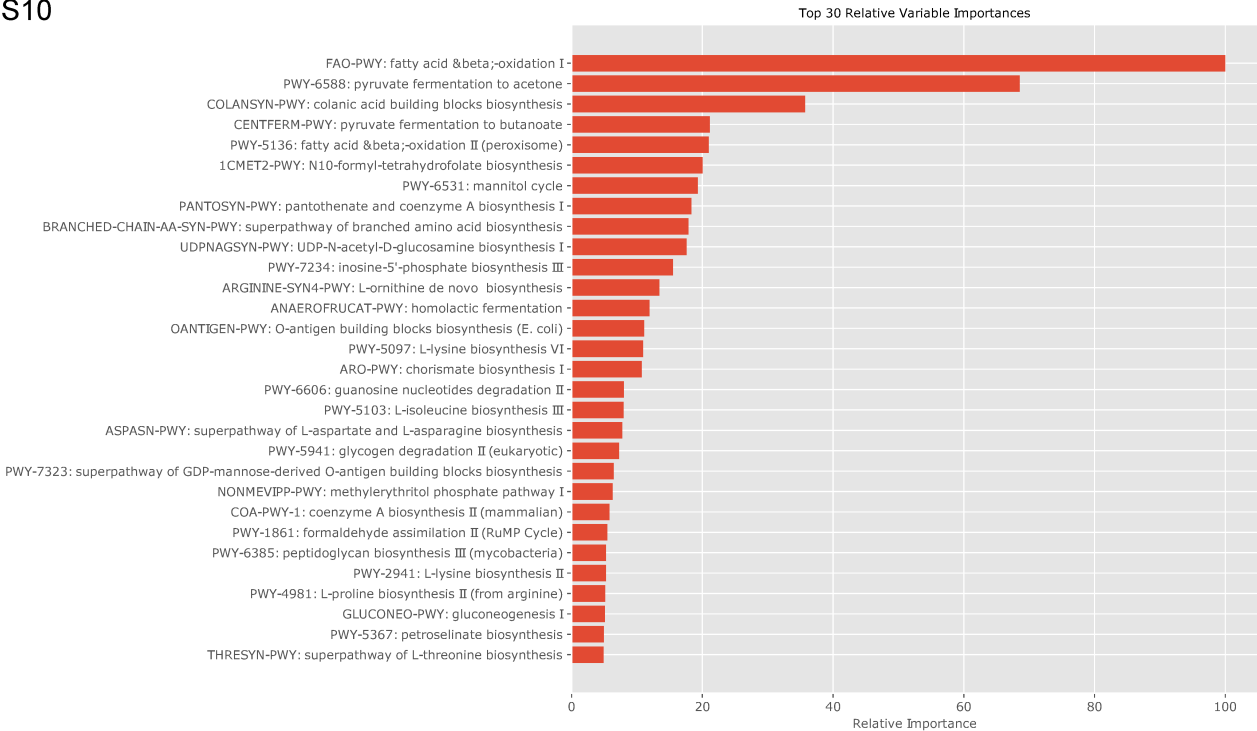

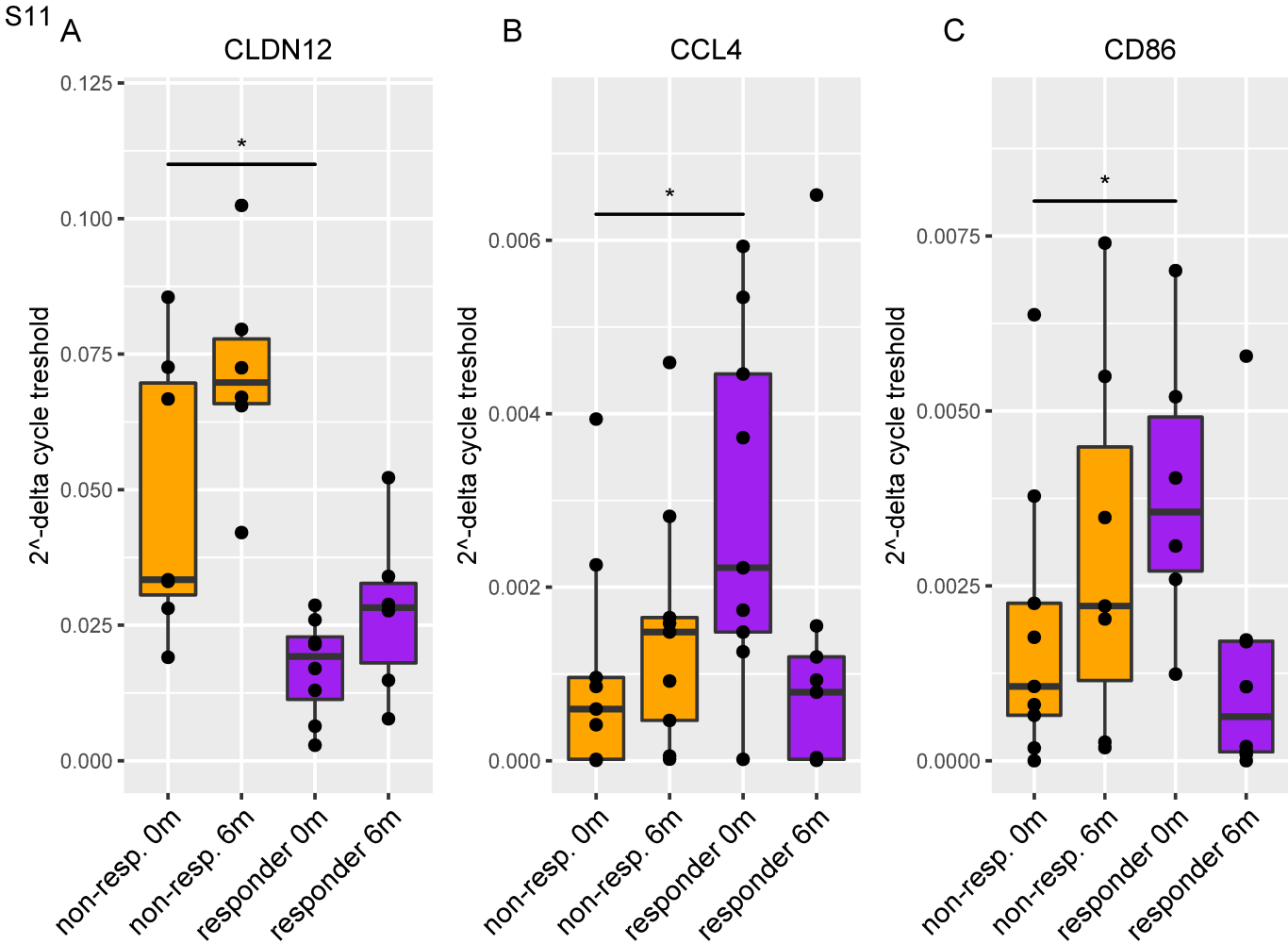

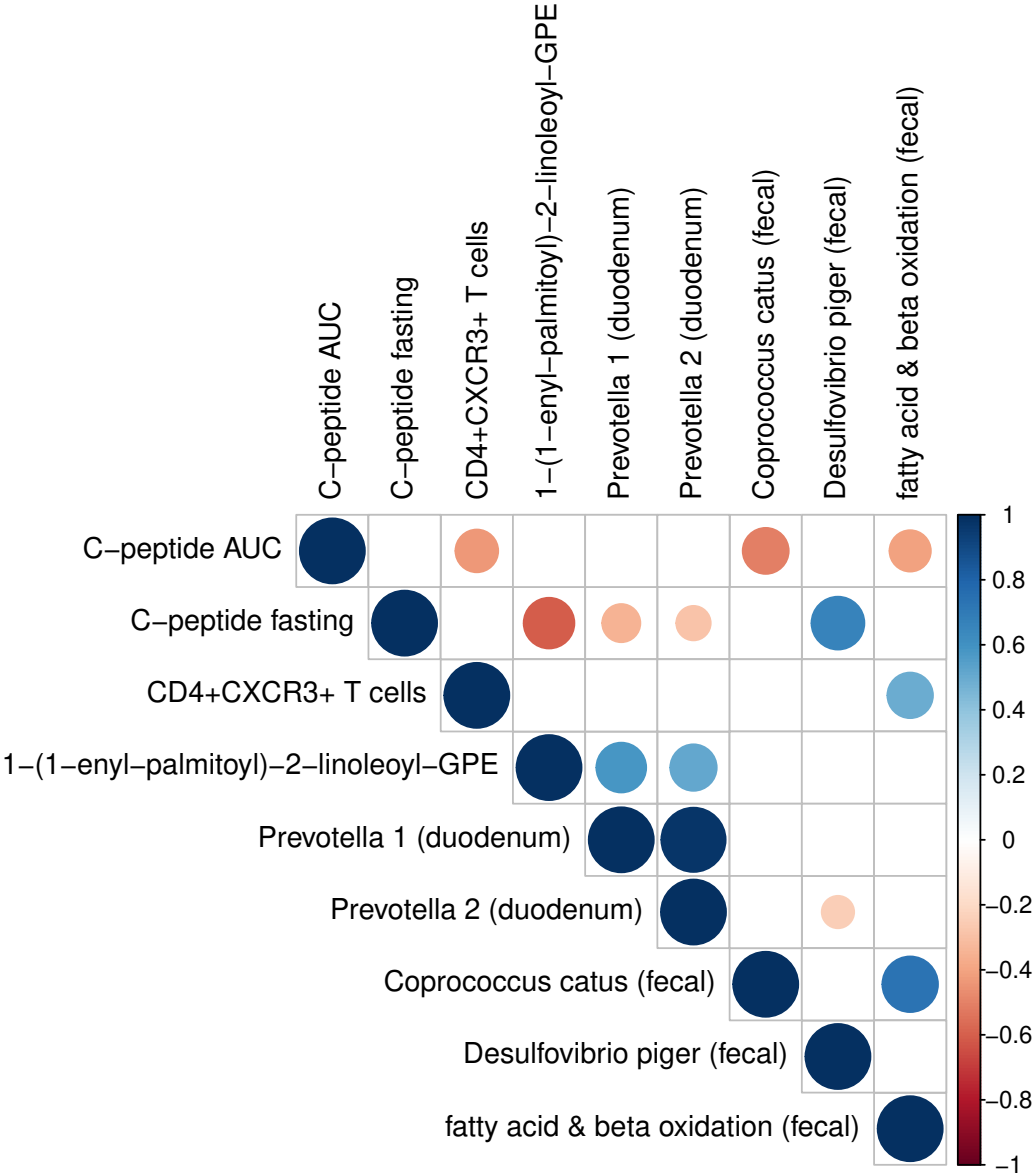

Supplement: Supplementary data [file gutjnl-2020-322630supp004.pdf]
