# Supplementary material for: Faecal microbiota transplantation halts progression of human new-onset type 1 diabetes in a randomised controlled trial
Source: Gut. 2020 Oct 26;70(1):92–105. doi: 10.1136/gutjnl-2020-322630 (PMC7788262; doi:10.1136/gutjnl-2020-322630)
Supplement: Supplementary data [file gutjnl-2020-322630supp005.pdf]

Cell type	Autologous (n=10)	Allogenic (N=10)	P value
Dendritic cells	17123	14529	0.07
Total monocytes	119555	73615	0.39
CD16 pos monocytes	7395	5539	0.07
CD14 pos monocytes	93804	72016	0.44
B cells	105975	172553	0.22
Naive B	61851	105175	0.22
non CS memory B	21187	20716	0.39
Transitional B	4463	3089	0.07
CS memory B	16577	21048	0.30
plasmablasts and plasmacells	3548	2826	0.07
NK cells	112375	123638	0.75
CD16 pos NK	95077	94477	0.82
CD56 NK	12090	18402	0.62
NKT cells	11571	11847	0.69
T cells	629591	588006	0.44
CD4 T pos cells	251710	228152	0.39
CD4 pos Naive T cells	120264	63899	1.00
CD4 pos CM	73353	46334	0.62
CD4 pos EM	36782	59531	0.75
CD4 TEMRA	7228	4172	0.50
CD4 pos B7 pos	5262	3544	0.34
CD4 pos CCR5 pos	11380	10425	0.15
CD4 CXCR3	39267	24162	0.06
CD8 pos	85578	67805	0.96
CD8 pos Naive	49335	28281	0.13
CD8 pos CM	7266	6906	0.34
CD8 pos EM	14732	6080	0.16
CD8 TEMRA	7688	5519	0.39
CD8 pos B7 pos	2413	1091	0.09
CD8 pos CCR5 pos	5141	3240	0.77
CD8 CXCR3	9237	3039	0.89
nTreg	8005	6190	0.30
Treg B7 pos	1070	339	0.96
Treg CCR5 pos	969	319	0.75
Treg CXCR3	847	303	0.62

Supplementary table 3: Number of Whole blood immune cells per group at baseline. p-values were calculated using Mann-Whitney U test.
